# Supplementary material for: The BrightEyes-TTM as an open-source time-tagging module for democratising single-photon microscopy
Source: Nat Commun. 2022 Dec 1;13:7406. doi: 10.1038/s41467-022-35064-0 (PMC9715684; doi:10.1038/s41467-022-35064-0)
Supplement: Supplementary file 1 — Supplementary Information [file 41467_2022_35064_MOESM1_ESM.pdf]

Supplementary Note 1: Sliding-Scale Technique and Auto-Calibration

The sliding scale approach is the most suitable FPGA-based TDC architecture for the BrightEyes-TTM as it incorporates crucial features for single-photon microscopy applications. In fact, the sliding-scale design allows a flexible multi-channel TDC implementation, which reduces the well-known non-linearity problem of FPGA-based TDCs and minimises FPGA resources, allowing the BrightEyes-TTM to be used with different laser sync rates and, moreover, granting the auto-calibration of the TTM.

The sliding-scale technique uses multiple flash TDC modules, one for each implemented channel (START channels), and a dedicated one for the STOP channel (i.e. the laser SYNC signal), deployed in the FPGA together with a coarse free-running digital counter (Suppl. Fig. S16). A key feature of this technique is the synchronous readout of the flash TDC and the coarse counter, together with the FPGA internal reference clock, which is totally uncorrelated with respect to the STOP signal and therefore also with the photon arrival time. The sliding-scale technique can thus be defined as an asynchronous design (1–3). An advantage of this design is that all the input signals (START_N and STOP_SYNC) produce time-tagging values over the full available dynamic range of the flash TDC. In short, the very same experiment can be used for calibration (e.g., to estimate for each delay unit of the flash TDC module the effective duration, Suppl. Note 3) independently of all flash TDCs, and to further compensate for the non-linearity.

Sliding scale technique. To tackle the linearity issue, the fundamental sliding scale TDC architecture is composed of a flash TDC module to sample START events (i.e. incoming photons) and a second flash TDC module to acquire the STOP signal (i.e. the laser sync signal) (Suppl. Fig. S17). As described in literature (4), a conventional FPGA-based flash TDC should not be used because of its lack of linearity across the probed temporal range, caused by intrinsic non-linearities in the FPGA fabric. The non-linear behaviour of flash TDC modules is shown in Fig S17 (a). In the sliding scale technique, instead of using a single flash TDC in which the delays between the STARTs and STOP signals are directly measured, two independent flash TDC modules are used in combination with a coarse counter. One flash TDC measures Δt_photon, i.e., the arrival time of the photon (START) with respect to an internal FPGA reference clock. The second TDC measures Δt_SYNC, i.e., the delay of the laser sync (STOP) with respect to the same FPGA reference clock, Fig S17 (a). The coarse counter is needed to calculate the time difference (Δt) between the photon and the laser pulse, Eq. S1 (see also Fig S17 a):

$$\Delta t = \underbrace{\left[n_\text{SYNC} \cdot \mathcal{T}_\text{sysclk} - \Delta t_\text{SYNC}\right]}_{t_\text{SYNC}} - \underbrace{\left[n_\text{photon} \cdot \mathcal{T}_\text{sysclk} - \Delta t_\text{photon}\right]}_{t_\text{photon}} \qquad \textbf{(S1)}$$

Since the photon and laser sync signal are asynchronous with respect to the FPGA clock, the same time interval Δt is measured in different regions of the flash TDC module for different photons. In the sliding scale approach, identical time intervals will therefore yield slightly different flash TDC responses. However, these non-linearities are averaged out over multiple observations (Suppl. Fig. S17 b). In order to reduce FPGA consumption (to further allow scaling up the current design) and to take advantage of the TTM auto-calibration system described below, the calculations and time conversions for computing Δt, Δt_photon and Δt_SYNC take place on a host computing unit during a post-processing phase. Here, the flash-TDC readings and the registered coarse counter values are merged for each channel to reconstruct the history of each photon (Suppl. Fig. S24). In the proposed TTM architecture, the conjunction of separate flash TDC modules and a common 16 bit-wide coarse free-running counter for all the implemented channels not only levels out the non-linearity but also offers the possibility to (i) keep track of the experiment time, allowing the TTM to have a virtually infinite temporal range, and (ii) to deal with different laser sync rates. Together with the photons and laser sync arrival times, the wrapping events of the coarse counter are also registered and used to calculate the experiment time by simply combining the number of wraps with the period of the FPGA reference clock (240 MHz i.e. ~4.2 ns). The ability of the TTM to reconstruct the time axis based on the internal FPGA clock also allows the TTM to be used with different laser sync frequencies without needing to change the FPGA firmware. As a result, the TTM can be used to work at different temporal ranges, having always the same linear response.

Auto-calibration. Thanks to the sliding-scale approach, the TTM calibration procedure can be carried out directly on the same experiment data. In order to calibrate a flash TDC module (i.e. assessing the time width of all the small delay elements that constitute a flash TDC, Suppl. Note 3) a statistical code density test needs to be performed (Suppl. Note 2). By sampling and accumulating random photon events, one can build up a look-up-table (LUT) that contains the time-conversion factors of all the possible TDC readouts to time-tag the incoming photons. In the sliding-scale approach, all the input signals (photons and laser sync events) are, by default, unsynchronized with the FPGA clock. The statistical code density test is thus built-in into the tagging architecture mechanism design: the TTM can be calibrated with experiment data, hence the name auto-calibration. All the deployed flash TDC modules are calibrated bin-by-bin in a post-processing phase using the reconstructed experiment data. In this way, voltage and temperature changes will not affect the data and timing reconstruction. In conclusion, although the auto-calibration is conducted off-line (i.e., not in real-time), it has the advantage that no ad-hoc calibration acquisitions are needed and, since it can be implemented on the computer, it does not need extra resources on the FPGA chip.

Bin-by-bin calibration. In the sliding scale approach, the flash TDC clock (FPGA clock) is not correlated with the $START_n$ or STOP inputs, i.e., all measured inputs have the same probability of falling into any bin of the tapped delay line of the flash TDC module (Suppl. Fig. S18 a). Since the bin widths (i.e. the delay element values of the tapped delay line) are not all equal due to the intrinsic FPGA irregularities, a photon or sync hit is more likely to fall into a wider bin than into a narrower bin (4), Fig. S27 (b) and Fig. S29. In order to calibrate the time response of the TTM, the histogram of the hit counts as a function of the arrival bin of each deployed flash TDC module is used. After having collected a large number of $START_n$ or STOP events from a measurement, the cumulative event count in each bin is proportional to its width. For example, if a total of N hits are accumulated into the histogram (Suppl. Fig. S27 a), assuming these hits are evenly spread over ~4.2 ns, which is the period of 240 MHz FPGA clock driving the flash TDC, then the width of an N_i-count bin is $w_i = N_i$*(4200 ps)/(N) (Suppl. Fig. S27 b). In the bin-by-bin calibration procedure, the widths w_i of all tapped delay line CARRY elements (Suppl. Note 3) are measured and stored in an array w_k, then the calibrated time responses Δt_i, corresponding to the center of i-th bin, can be calculated according to Eq. S2 (5, 6):

$$\Delta t_{\mathrm{i}} = \frac{w_i}{2} + \sum_{k=0}^{i-1} w_k \tag{S2}$$

In this way, all the different Δt_i time contributions of all the flash TDC module delay elements, can be assessed and used to correctly time-tag both the photons ($START_n$) and the laser sync line (STOP) (Suppl. Fig. S27 c).

TCSPC histogram binning. Since we use the sliding-scale (or Nutt) method to implement the fine TDC, and an off-line (post-acquisition) bin-by-bin calibration, the user can arbitrarily choose the bin width of the start-stop (TCSPC) histogram. In this work, we always used the same bin width value, i.e., 48 ps. We used this value for the code density test (i.e., the least-significant-bit (LSB) is equal to 48 ps), for the single-shot precision measurements, and for all the experimental measurements. This value is much lower than the system IRF full-width-at-half maximum (200 ps), and it is in the same range of the CARRY's average temporal length, i.e., the length of the tapped delay elements used to implement the TDC. Supplementary Fig. S29 shows the calculated (bin-by-bin calibrated) w_k bin widths for all the N = 25 deployed flash-TDC modules in a typical code-density experiments. The average values for the bin widths is (43 ± 16) ps. Notably, this information is not used in any TCSPC histogram reconstruction, since the bin width is arbitrary chosen by the user. Supplementary Figure S28 shows the very same start-stop histogram (i.e., the same experiment) for different bin-width values. The histogram's shape changes substantially only for bin-widths higher than the width of the system IRF. This value is lower than the system IRF full-width-at-half maximum (200 ps) and in the same range as the CARRY's temporal length. The average of the calculated (bin-by-bin calibrated) w_k bin widths for all the N = 25 deployed flash-TDC modules is 48 ps. For this reason, 48 ps is chosen as the LSB value and used as the standard bin width to create the photon count histograms. Since we use the Nutt-sliding scale module and off-line post-acquisition calibration, the bin width can be virtually chosen to be any value greater than 48 ps (Suppl. Fig. S28).

Supplementary Note 2: Statistical Code Density Test

The code density test allows measuring (i) the time response of each time-bin of the TDC and (ii) the deviation of TTM measurement readouts from the actual time of arrival of a photon. One of the most important parameters to analyse in order to asses the system response of a time-tagging device is the system linearity. A statistical code density test consists of feeding the TTM random (uncorrelated) photons with respect to the sync reference signal. Here, we generated random photons by connecting an avalanche photodiode (APD) to the TTM in a no-light room (Suppl. Fig. S22). After reconstructing the histogram $H_{(i)}$ of the collected random events (using bin-by-bin auto-calibrated data), $H_{(i)}$ histogram data is used to compute two benchmark indicators of a system's linearity: the differential non-linearity (DNL) and the integral non-linearity (INL) (Suppl. Fig. 2 a). The DNL and INL may seem counter-intuitive indices as the DNL describes the non-linearity amongst the different time-bins of $H_{(i)}$ (i.e., how much all the bin widths in the temporal range differ from each other) and the INL to what extent the system is non-linear (i.e., to what extent the system is capable of precisely measuring time with respect to an ideal time-tagging device). The relevant values are the standard deviations of the DNL and INL, which are expressed in LSB. In agreement with the Xilinx Kintex-7 XC7K325T-2FFG900C datasheet, an LSB of 48 ps, corresponding to the time delay associated with the coarseness of the CARRY4 element (employed as the fundamental unit in the flash TDC module) was used to reconstruct $H_{(i)}$ and compute σ_{DNL} and σ_{INL} (7).

DNL - Differential non-linearity. When performing a statistical code density test, the reconstructed time histogram $H_{(i)}$ should ideally be a constant flat line, indicating that every time-bin has an equal time-width within the measured temporal range. In reality, due to intrinsic FPGA fabric inconsistencies, $H_{(i)}$ shows a ripple that needs to be characterised in order to maximise the timing precision and accuracy. The aim of computing the DNL is to understand to which degree the difference in time widths of all the possible time bins (which is the cause of the ripple) deviates from a common average value. In other words, the DNL is used to understand the relative contribution of all the time bins when reconstructing times of arrival of photons in the TDC temporal range. The DNL is calculated according to Eq. S3:

$$DNL_{(i)} = \frac{H_{(i)} - H_{\text{avg}}}{H_{\text{avg}}} \tag{S3}$$

Here, $H_{(i)}$ is the reconstructed histogram of the dark counts and H_{avg} its average value. $DNL_{(i)}$ represents the deviation of the i-th time bin from the H_{avg} value: the lower this deviation, the flatter the $DNL_{(i)}$ plot will be. If the DNL is constant, all time-bins have the same width and the system is perfectly linear. Having set the LSB to 48 ps, σ_{DNL} is about 6 % of the LSB, yielding an RMS value of 2.88 ps: the average time width of the histogram bins is thus: (48 ± 3) ps.

INL - Integral non-linearity. The INL is used to determine to which degree the response of a time-tagging system differs from the ideal linear behaviour. The INL gives an estimate of the difference between a TTM measurement and the actual time of arrival of a photon. If the TTM is linear, the INL is constant and zero.
The $INL_{(i)}$ is computed as the cumulative sum of all the $\text{DNL}_{(i)}$ contributions, Eq. S4:

$$INL_{(i)} = \sum_{k=1}^{i} DNL_{(i)} \tag{S4}$$

The σ_{INL} for all the TTM channels is 8 % of the LSB, which corresponds to an RMS value of 3.84 ps.

Supplementary Note 3: Flash TDC module

In this supplementary section, we describe the principal components and the associated functions that constitute the flash TDC module used in the TTM architecture. The core constituent of a flash TDC module is a tapped delay line (TDL). The TDL consists of a series of small delay elements joined in a chain architecture and is used to delay an input (START) signal with respect to a reference-sampling FPGA digital clock. From the delay characteristics of each delay element, it is possible to match the distance covered by the START signal along the delay line with a specific arrival time (time-tag) with respect to the FPGA clock signal (8, 9), Fig S17 a.

Tapped Delay Line and latch barrier. A specific Xilinx FPGA primitive function block known as CARRY (CARRY4 or CARRY8 depending on the FPGA family) was used as a fundamental delay block to build the tapped delay line, formed by connecting multiple CARRY blocks. Each delay block is connected to a latch component that can retain either a boolean '0' or '1' depending on the data input value at the rising-edge of an FPGA clock. Assuming that the START signal is '0' (false) at the steady state and '1' (true) after a triggering event, the '1' value will propagate through the tapped delay line elements and, consequently, the data input of each latch (bit_n) will also change from '0' to '1'. The START signal propagates freely until a rising-edge of the FPGA clock occurs. This rising-edge freezes the values of the latch barrier connected to the TDL elements giving out a digital reading of how far the START signal travelled along the TDL within the FPGA internal clock period. Knowing the reciprocal relationship between the CARRY delay value (and also knowing the delay contribution of each CARRY element in the chain) and the distance the START signal covered before the rising-edge of the FPGA clock occurred, it is possible to convert the digital measurement of the travelled distance into a time measurement (10), Suppl. Fig. S18 a.

Thermometer to binary encoder. The readout from the latch barrier is a series of '1' and '0' that have to be converted into a binary value for a more efficient representation. Therefore, a dedicated circuit is needed to interpret and decode the TDL data. The thermometer-like readout coming from the latch barrier is sent to a thermometer to binary encoder component (T2B). The T2B accepts an array of n-bits as input and returns a binary number that represents how many '1' are present in the input latched data. This T2B conversion simplifies the TDL readout, allowing for a more effective data registration in terms of memory resources utilisation (11), Suppl. Fig. S18 b.

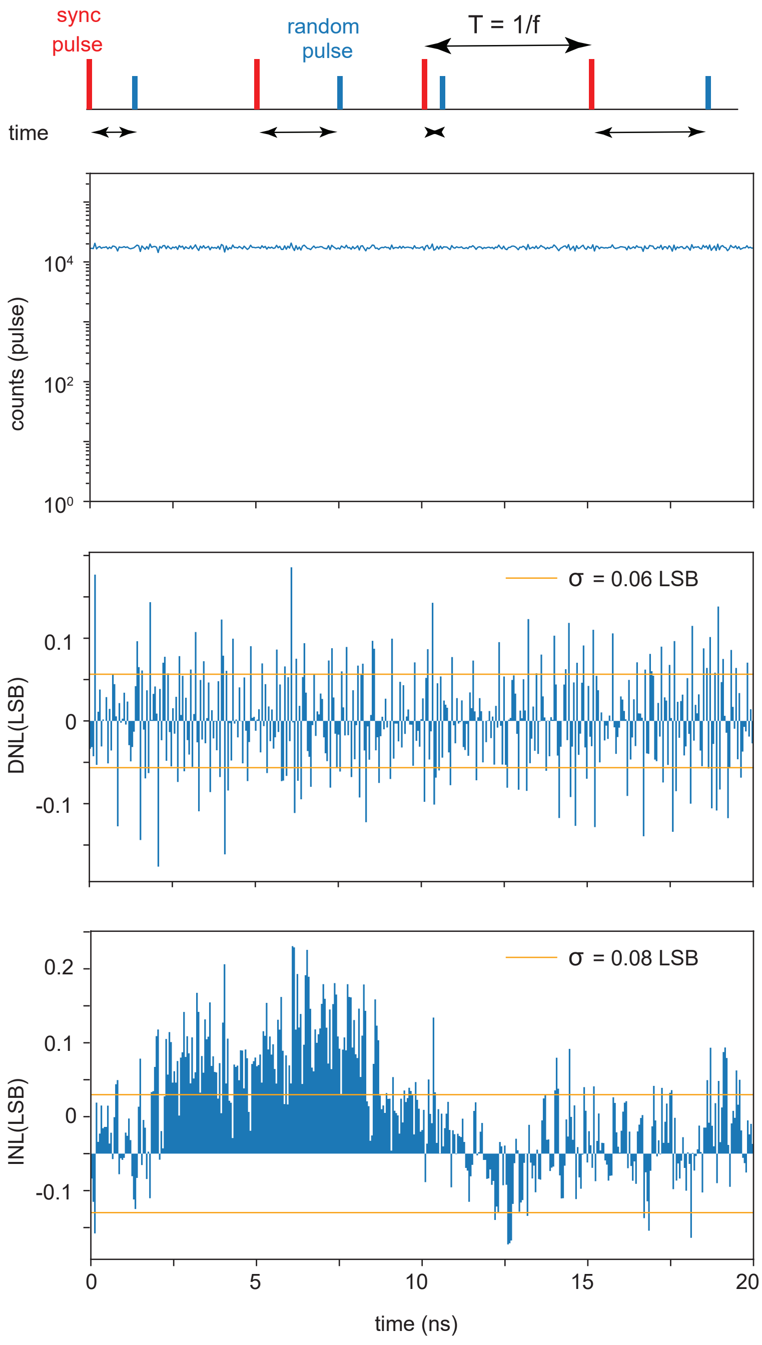

Fig. S1. Statistical code-density test. Schematic representation of the signals involved in the statistical code-density experiment: a fixed frequency SYNC (STOP) clock signal at f = 50 MHz (i.e, $\mathcal{T} = 1/f$ = 20 ns) and an uncorrelated/random train of pulses (START). The reconstructed start-stop time histogram, i.e., counts versus time (top), the relative differential non-linearity (DNL) (middle) and the integral non-linearity (INL) (bottom).

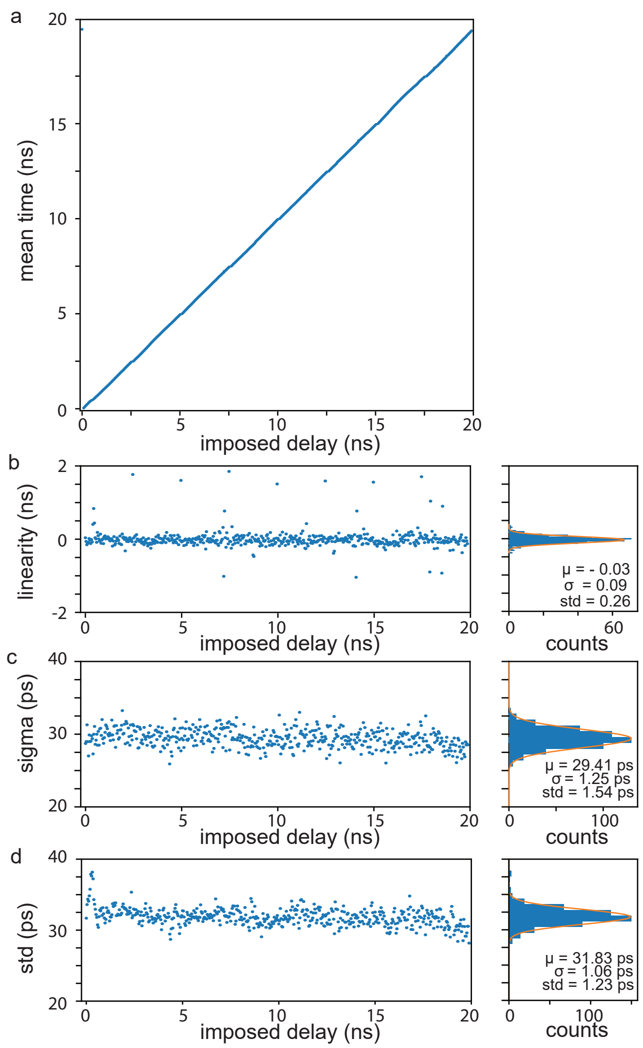

Fig. S2. Single-Shot Precision Measurement. Schematic representation of the single-shot precision experiment for channel #12. The experiment uses the BrightEyes-TTM to repeatedly measure the start-stop interval between a fixed 50 MHz signal, used as SYNC signal, and a synchronised second signal, used as photon signal. The experiment is repeated for all possible delay values (imposed delay) within the repetition rate of the SYNC signal. For each imposed delay value, we collected several millions of sync-photon pairs, and built the start-stop time histogram (not shown). Each single histogram is fitted with a Gaussian distribution $A\exp\{-((t-\mu)/\sigma)^2/2\}$ to extract the mean μ and the standard deviation σ. **a** The mean value μ as a function of the imposed delay. **b** The difference between the imposed delay and the mean value obtained as function of the imposed delay. **c** The standard deviation σ value as function of the imposed delay. **d** Calculated standard deviation of the start-stop time histogram as a function of the imposed delay. The similar values between the Gaussian standard deviation σ and the calculated standard deviation demonstrate the normal distribution of the start-stop time histogram. The relevant statistics are reported on the right-side of each graph.

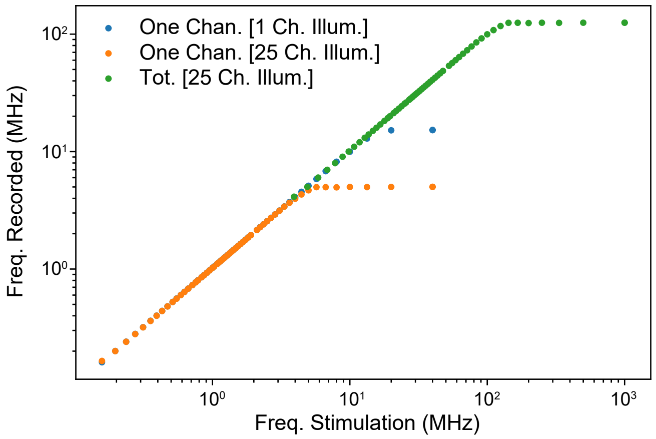

Fig. S3. Sustained count rate. The BrightEyes-TTM inputs channel were connected to the SYLAP signal generator. The plots shows that the TTM can sustain an overall count rate (green) of about 125 MHz when all 25 channels are simultaneously excited, limiting the count rate per channel to about 5 MHz (orange). If only a single channel is stimulated, the maximum count rate may be up to around 15 MHz (blue). Notably, in this work we used SPAD array detectors with 100 ns hold-off time, thus already limiting the sustained photon rate for each channel to 10 MHz.

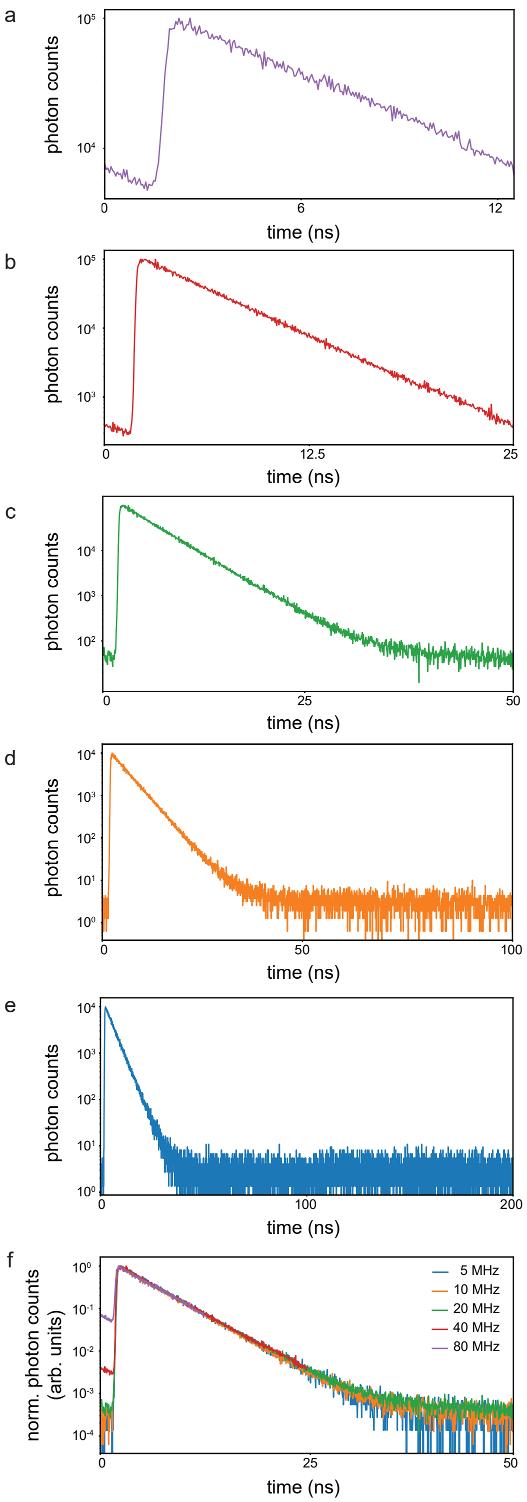

Fig. S4. Validation of the TTM for different temporal ranges (a-e) and comparison of the obtained results (f). Fluorescence decay histogram (photon counts as a function of time) of a fluorescein-water solution for **a** 80 MHz, **b** 40 MHz, **c** 20 MHz, **d** 10 MHz and **e** 5 MHz laser repetition rates. **f** Cumulative view (normalized photon counts versus time) of the reconstructed fluorescein decay histograms for all the probed temporal ranges. 40 MHz and 80 MHz curves show a higher offset when compared with 5, 10, and 20 MHz plots, due to a non-complete relaxation of the fluorescein molecules from the excited state that occurs with shorter laser excitation periods. Setup: custom-built single-photon laser scanning microscope equipped with a 5×5 SPAD array detector prototype. Data from central element only.

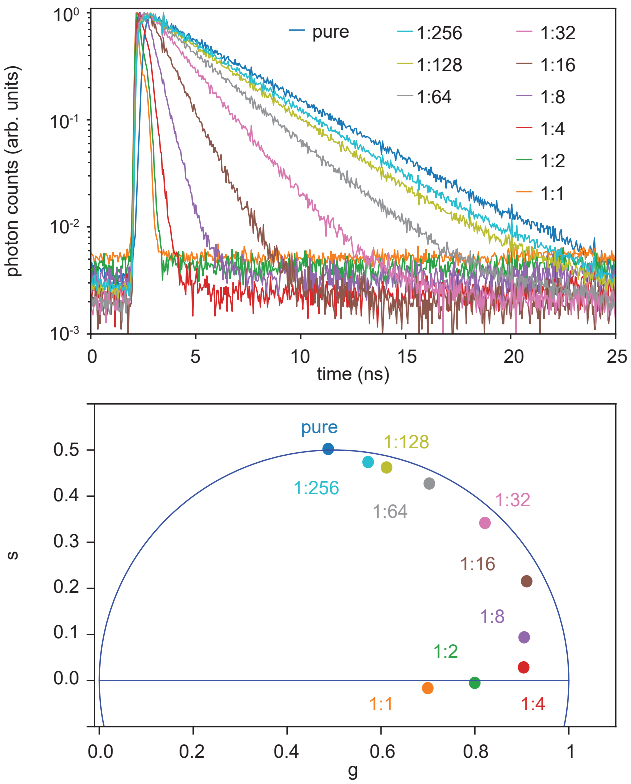

Fig. S5. Quenched fluorescein solutions. Normalised fluorescence lifetime decay histograms of quenched fluorescein solutions for increasing concentrations of quencher (potassium iodide) (top), phasor representation of quenched fluorescein solutions (bottom). All single-channel measurements were done with TTM channel #12, which received the photon signal from the central element of the SPAD array detector. All fluorescence lifetime decay histograms have 48 ps granularity (bin-with). Setup: custom-built single-photon laser scanning microscope equipped with a 5×5 SPAD array detector prototype.

Channels	**21**	**25**	**49**
**LUT**	3.5%	3.8%	5.8%
**LUTRAM**	0.9%	0.9%	1.1%
**FF**	3.8%	4.2%	6.9%
**BRAM**	15.4%	17.0%	26.5%
**IO**	20.2%	26.0%	35.6%
**BUFG**	25.0%	25.0%	25.0%
**MMCM**	20.0%	20.0%	20.0%
**Total occupancy**	12.7%	13.8%	17.3%

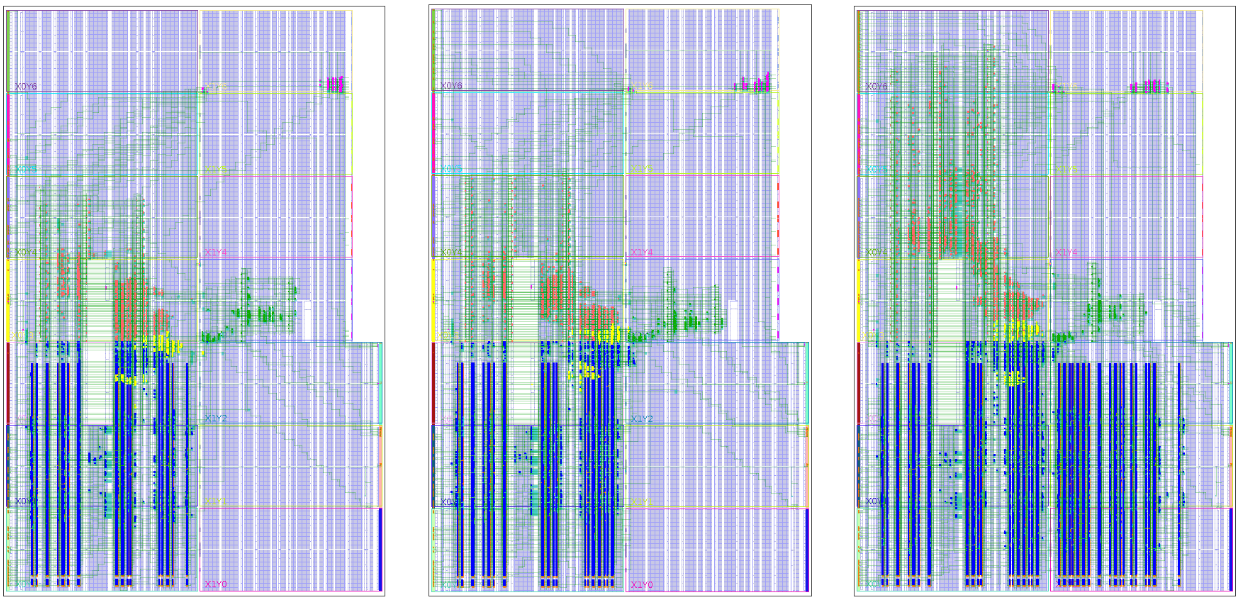

Fig. S6. FPGA resource utilisation. The three maps show the FPGA resources with firmware designed for 21 (left), 25 (central) and 49 (right) channels; the tapped delay line (blue), the FX3 module (green), the data preparation module (red), SYLAP (magenta, only for test purposes), and the ILA debugger (yellow). The percentage of FGPA resources occupancy is given in the above table. The acronyms follow the standard Xilinx 7-Series names: look-up-table element (LUT); random memory access implemented on a look-up-table (LUTRAM); flip-flop (FF); dedicated block-ram (BRAM); input/output pins (IO); global FPGA buffer (BUFG) and Mixed-Mode Clock Manager (MMCM).

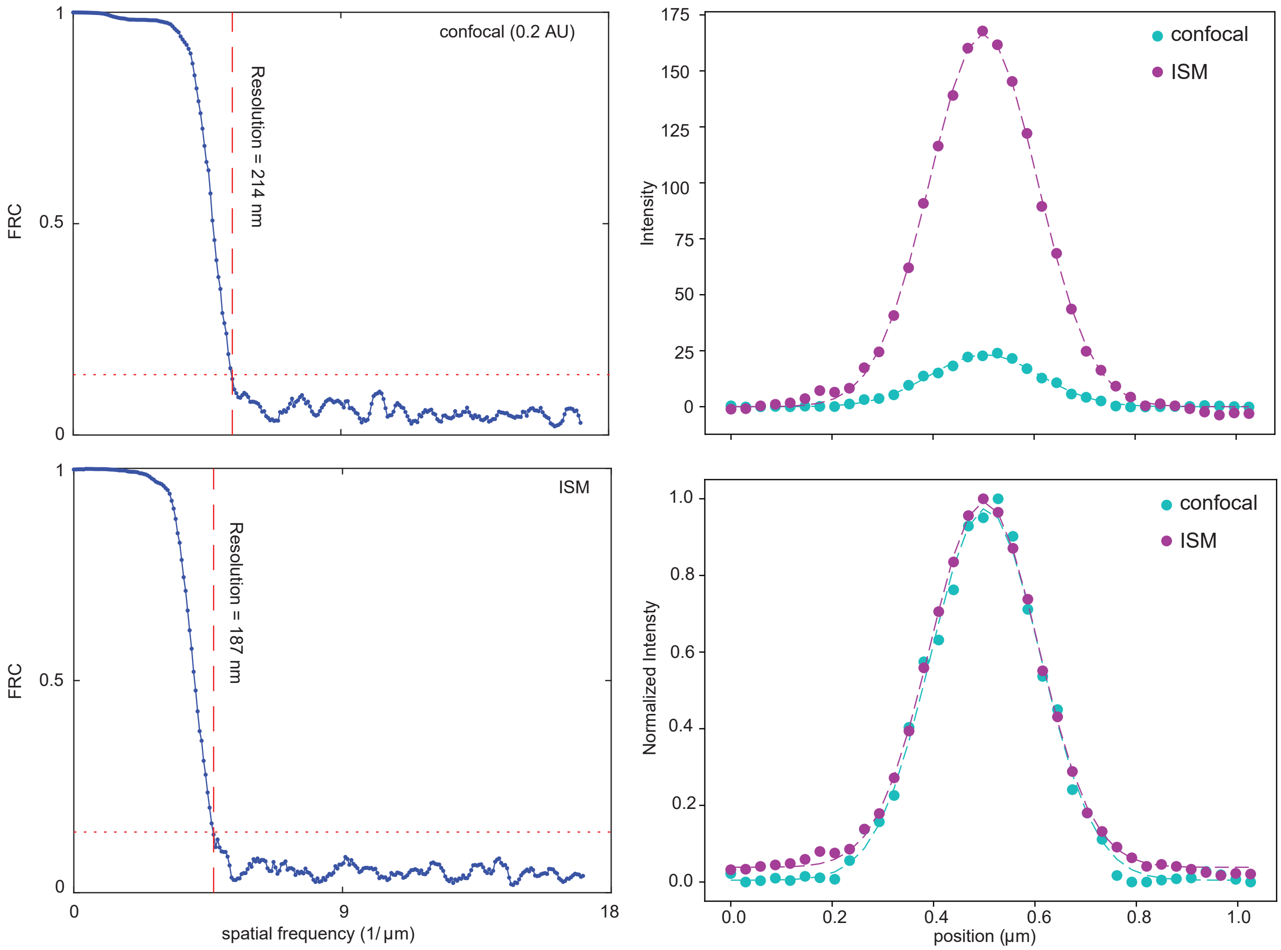

Fig. S7. Fourier ring correlation (FRC) analysis and intensity line profile for confocal and ISM images of YG carboxylate fluoSpheres. The panels show the FRC analysis for the confocal (0.2 AU) images (top, left) and the APR-ISM images (bottom, left). Each curve represents the decay of the correlation as a function of the spatial frequency. We used the $1/7$ criteria (short dotted red lines) to estimate the effective cut-off frequencies of the images. The inverse of these values, which are reported in each graph, represents the effective spatial resolution of the images. On the right, the line profiles of the fluorescence intensity of the same fluoSphere for the confocal (0.2 AU) image and the APR-ISM image. As expected, the signal-to-noise ratio significantly improves in the APR-ISM case compared to confocal (top), while the optical resolution (i.e., the full-width-at-half-maximum is similar in both cases (bottom). Importantly, in case of fluorescence lifetime imaging, an increase in SNR leads to a more precise estimation of the fluorescence lifetime.

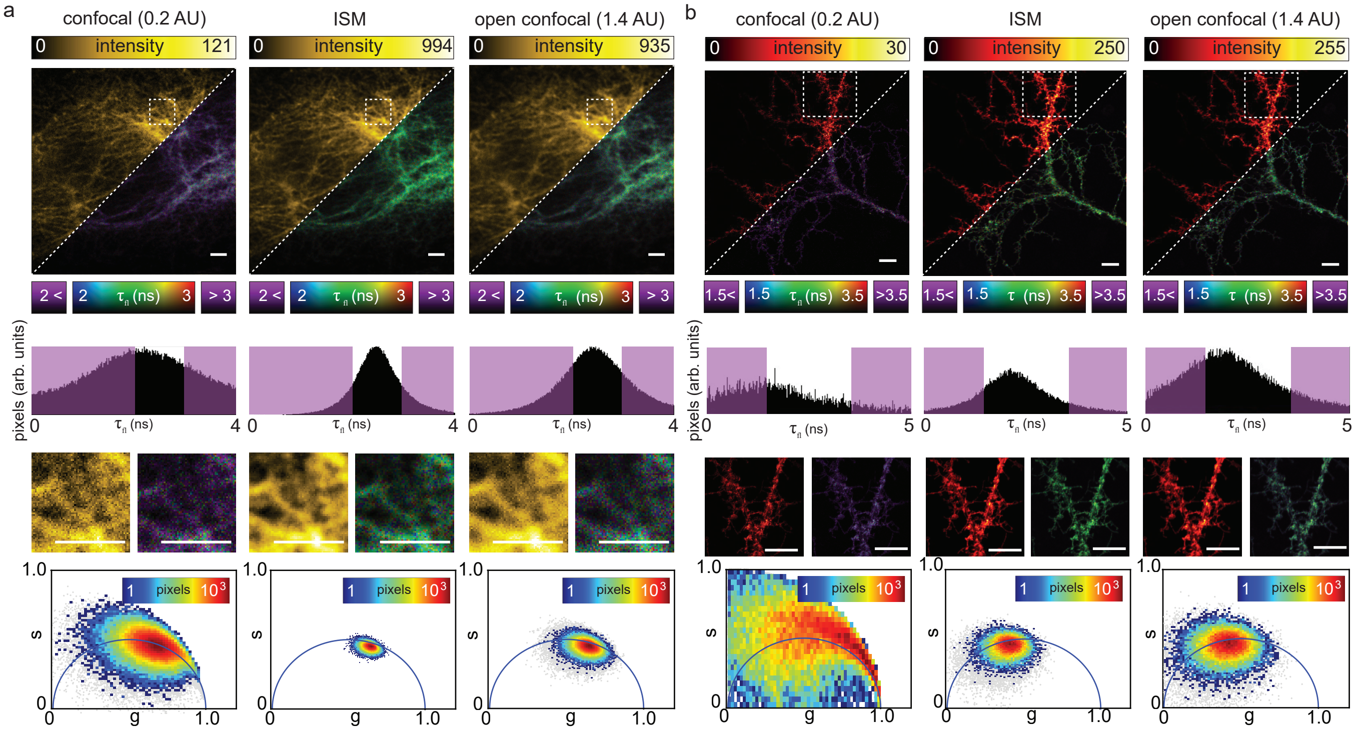

Fig. S8. Imaging and FLISM analysis of biological samples. **a** Imaging and analysis of fluorescently labelled vimentin in fixed cells. Side-by-side comparison (top row) of confocal (left, pinhole 0.2 AU), adaptive pixel-reassignment ISM (center), and open confocal (right, pinhole 1.4 AU). AU = Airy unit. Each imaging modality shows both the intensity-based image (top-left corner) and the lifetime image (bottom-right corner). The bi-dimensional look-up-table in the lifetime images represents both the intensity values and the lifetime values. Histogram distributions of the number of pixels versus lifetime values (middle-top row) - in violet lifetime values which fall out of the selected lifetime interval. For **a**, the lifetime histogram bounds are symmetrical around the peak in the FLISM histogram. The width was chosen by visually inspecting the FLISM image and maximizing the contrast. The same bounds were used for the confocal and open confocal data. For **b**, the histogram bounds were chosen by visually inspecting the FLISM image and applying the same bounds to the confocal images. The lifetime images report in violet the pixels whose lifetime belongs to this interval. Zoomed regions in the white-dashed boxes, the intensity panels are re-normalised to the maximum and minimum values (middle-bottom). Pixel intensity phasor plots (bottom), 5% and 10% thresholds respectively in grey and color. Scale bars 2 µm. Pixel-dwell time 100 µs. **b** Imaging and analysis of live primary mouse neurons expressing (SEP)-tagged-$\beta 3$ subunit of the $GABA_A$ receptors. Images and graphs appear in the same order as described for **a**. Scale bars 10 µm. Pixel-dwell time 200 µs. The data were acquired n both samples from different cells, in particular for **b** two independent experiments with primary mice neurons were performed. Here, one image is chosen as representative example of the experiment. Setup: custom-built single-photon laser scanning microscope equipped with a 5×5 SPAD array detector prototype.

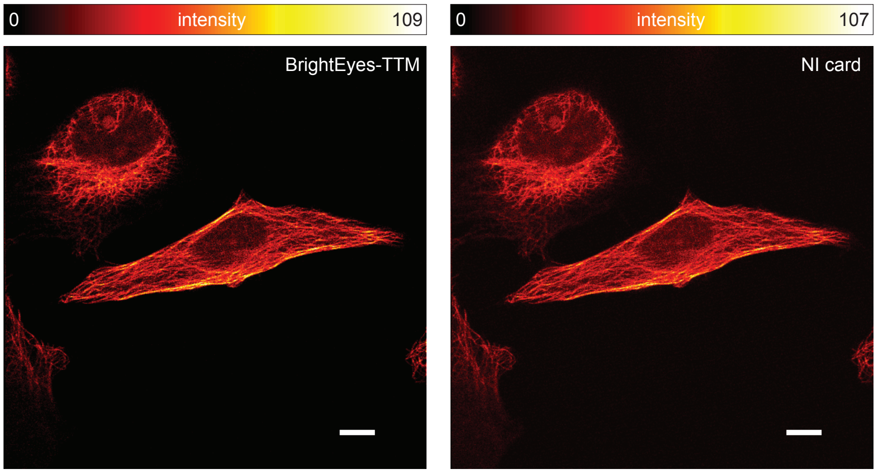

Fig. S9. Imaging with the BrightEyes-TTM and the real-time NI-DAQ system. Side-by-side comparison of confocal (0.2 AU) images obtained with **a** our TTM and **b** the NI-DAQ systems. The images represent α-tubulin immunolabelled Hela cells. The images were collected simultaneously: the BrightEyes-TTM received the signal from the central element of the SPAD array detector, duplicated it, and sent one copy back to the NI-DAQ system, while using the original signals to create the time-tagged data-set. The NI-DAQ system generated in real-time the confocal images, whilst the TTM image was generated off-line. Scale bars 15 µm. Pixel dwell time 200 µs. This comparison was performed only once. Setup: custom-built single-photon laser scanning microscope equipped with a 5$\times$5 SPAD array detector prototype.

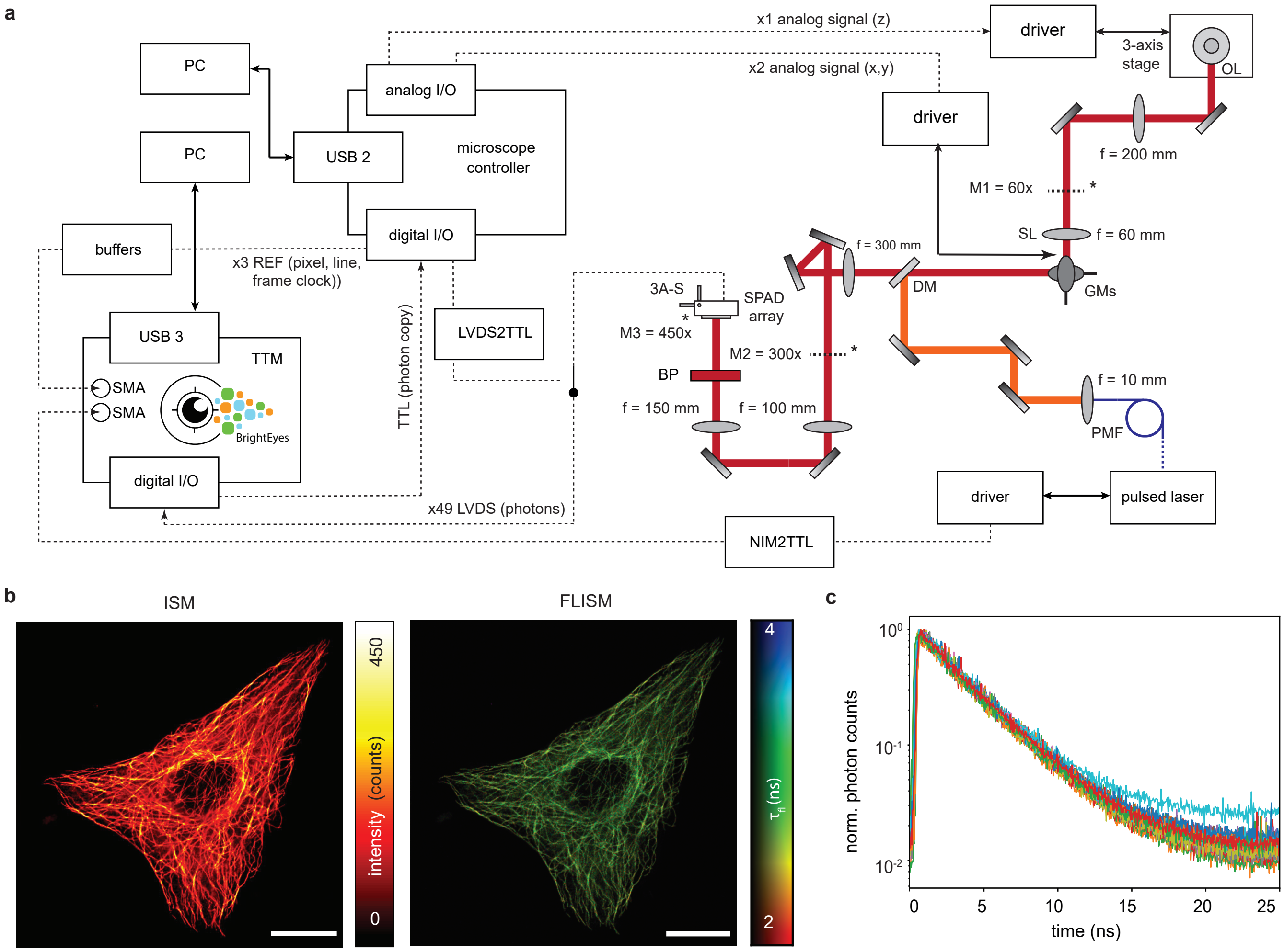

Fig. S10. Imaging and spectroscopy with a commercial 7×7 SPAD array detector. a Schematic representation of the optical architecture, the data-acquisition and control system. DM: dichroic mirror, GMs: galvanometric scan mirrors, SL: scan lens, TL: tube lens, OL: objective lens, BP: band pass filter, PMF: polarising maintaining fiber, PC: personal computer. The focal length of all lenses is reported in the figure. The asterisks depict the (intermediate) image planes, together with the magnifications. The microscope uses a 640 nm triggerable pulsed (~80 ps pulse width) diode laser (LDH-D-C-640). After being reflected by a multi-band dichroic mirror (ZT 405-488-594-640-775, Chroma), the excitation beam is deflected by two galvanometric scanning mirrors (6215HM40B; CT Cambridge Technology) and directed towards the objective lens (CFI Plan Apo VC 60×/1.4, oil, Nikon) by the same set of scan and tube lenses used in a commercial scanning microscope (Confocal C2; Nikon). The fluorescence light is collected by the same objective lens, descanned, and passed through the multi-band dichroic mirror and then through a suitable fluorescence band-pass filter (685/70; AHF Analysentechnik). A 300 mm aspheric lens (Thorlabs) focuses the fluorescence in a conjugated image plane with a magnification of 300×. A telescope system consisting of two aspheric lenses of 100 mm and 150 mm focal length (Thorlabs), conjugates the SPAD array with the pinhole and provides an extra magnification factor. The final magnification in the SPAD array plane is 450×, and thus the size of the central 5×5 SPAD array element projected on the specimen is ~1.4 AU (at the far-red emission wavelength, i.e., 650 nm). Notably, only the inner 5×5 central detector elements of the 7×7 SPAD array detector are registered by the system. Digital and analogue single-cable connections are represented by dashed lines. The pixel, line, and frame reference signals and the laser sync signal are directly plugged into the FPGA-development board with the SMA user I/Os, whilst the photon signals are connected to the board through the I/Os daughter card. The board duplicates the photon signal from the central element of the SPAD array detector and sends it back to the microscope controller via a TTL digital signal. Because the commercial detector output signals have a LVDS standard and the microscope control card (i.e., the FPGA-based National Instruments data-acquisition card) can read only TTL signals, when running the microscope in counting mode (i.e., the BrightEyes-TTM is not used), the outputs of the commercial SPAD array detector are converted to TTL (PRISM Light detector TTL outputs, Genoa Instruments). **b** ISM and FLISM imaging. The images represent an α-tubulin immunolabelled Hela cell. Adaptive pixel-reassignment ISM (left) and FLISM analysis (right). Scale bars 10 µm. Pixel dwell time 150 µs. **c** Start-stop histograms for the different channels of a solution of Atto-633NHS in water. This experiment was independently performed once, 3 different field-of-views were acquired.

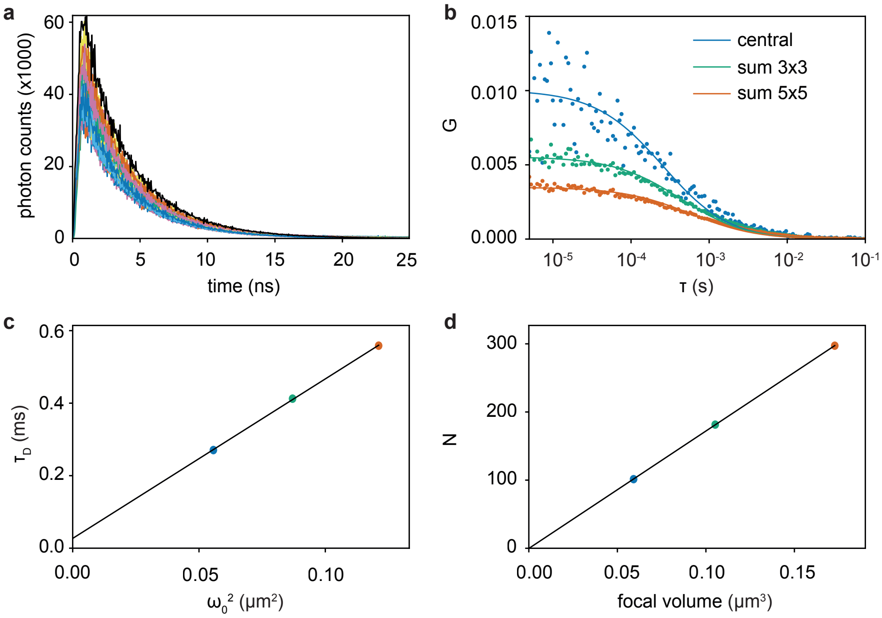

Fig. S11. BrightEyes-TTM for FLFS on freely diffusing goat anti-mouse antibodies conjugated with Alexa-488. **a** Start-stop time histograms, bin width 48 ps, central pixel in black. **b** (unfiltered) autocorrelations. **c** Diffusion time as a function of ω_0^2. The corresponding diffusion coefficient is (53 $\pm$ 2) μm^2/s. **d** Average number of particles in the focal volume as a function of the focal volume. The corresponding particle concentration is (1720 $\pm$ 4) /μm^3. Results are averages and standard deviations over 3 measurements of 130 s each. Here, the sample and the experimental conditions (concentration and laser power) lead to start-stop time histograms without a significant uncorrelated background. As a result, the correlation curves do not need filtering, and both the diffusion time and the average number of particles in the focal volume follow the expected behaviour. Setup: custom-built single-photon laser scanning microscope equipped with a 5$\times$5 SPAD array detector prototype.

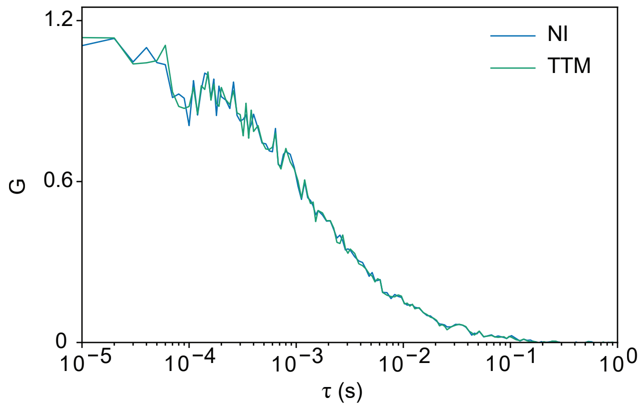

Fig. S12. Single-spot fluorescence correlation spectroscopy with the BrightEyes-TTM and NI-DAQ system. Comparison of the two autocorrelation curves obtained by recording simultaneously the signal with the two different platforms. The autocorrelation curves were calculated on the signal collected from the central element of the SPAD array detector in a sample of freely diffusing fluorescent beads. Average over 13 traces of 10 s each. Setup: custom-built single-photon laser scanning microscope equipped with a 5×5 SPAD array detector prototype.

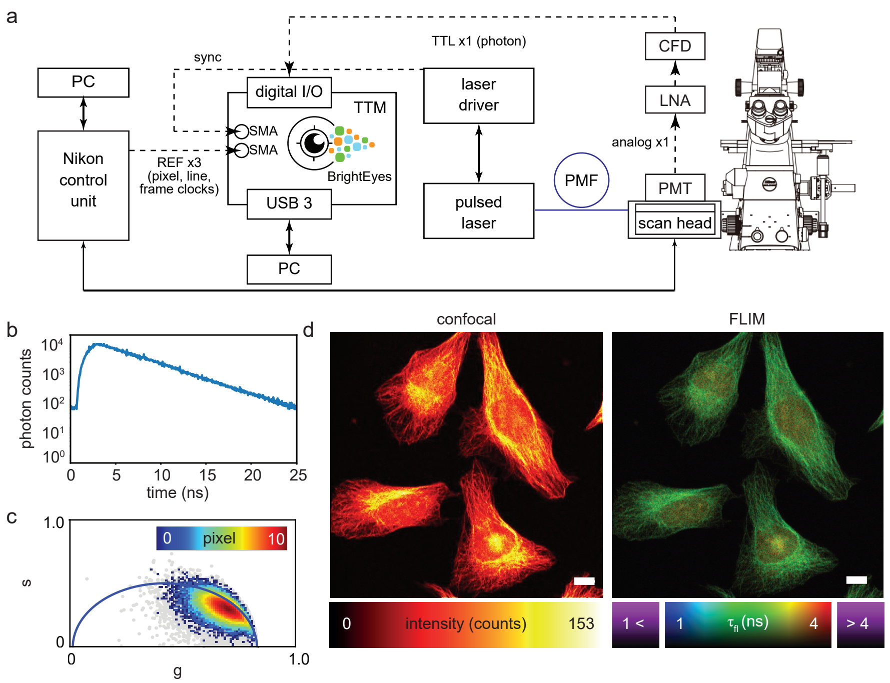

Fig. S13. Fluorescence lifetime measurements and FLIM with the BrightEyes-TTM connected to a commercial setup. a Experimental setup. We connected the BrightEyes-TTM to a Nikon A1 confocal microscope. PMT = photomultiplier tube, PMF = polarising maintaining fiber, CFD = constant fraction discriminator, LNA = low-noise amplifier. **b** Start-stop histogram of a coumarin solution. Fitting the histogram with a one component exponential decay yields a lifetime of 4.28 ns. **c,d** Images (**c**) and corresponding phasor plot (**d**) of Hela cells with α-tubulin immunolabelling, intensity-based (left) and lifetime based (right) image. Both approaches, phasor-based and fitting-based, can be accessed with our system. Scale bars 10 μm. Pixel dwell time 107 µs.This experiment was independently performed once on this setup.

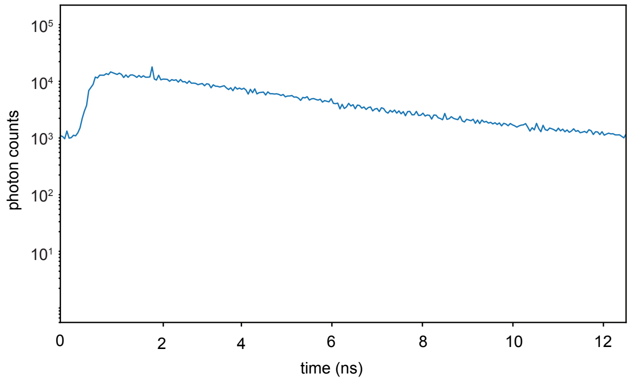

Fig. S14. Start-stop histogram of a fluorescein-water solution with the BrightEyes-TTM on a custom-built setup with a commercial single-photon detector. Fluorescence decay histogram (photon counts as a function of time) of a fluorescein-water solution measured with the BrightEyes-TTM on the custom-made confocal setup described in the Method section employing a commercial single photon detector ($PD-050-CTC-FC, Micro Photon Devices, Bolzano, Italy).

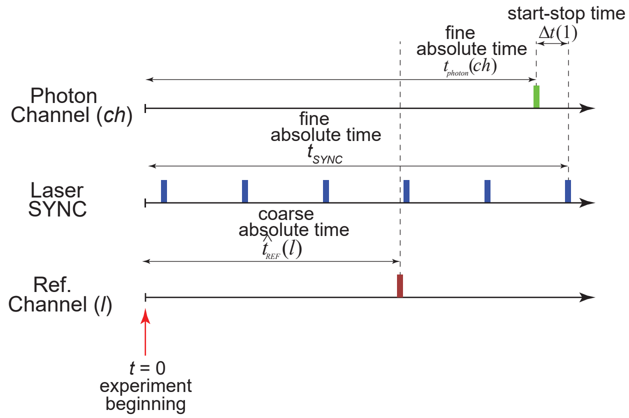

Fig. S15. Time-Tagging Principle. The time-tagging mode allows recording individual events and labelling each one of them with a temporal signature. Typically, this temporal signature denotes the time delay of the event with respect to the beginning of the experiment (absolute time). Our TTM is able to tag three different classes of events: the photon events, i.e., a photon is registered by the detector; the sync laser events, i.e., the synchronisation signal delivered by a pulsed laser; the reference events, i.e., a signal generated by another component of the setup (e.g., an actuator, a laser modulator). Each class of events reports the temporal signature with a different precision: the absolute times for the photon events (t_{photon}) and the laser synchronisation events (t_{SYNC}) have picosecond range precision, while the absolute time for the reference events ($\hat{t}_{\text{REF}}$) has nanosecond range precision. Starting from these temporal signatures, it is possible to derive more information. For example, for each photon event, one can derive the so-called start-stop time (Δt), which describes the delay of the photon event with respect to the successive laser sync event. Table S1 in the Supplementary describes the main temporal signatures used in this work. Importantly, together with the temporal signatures, the TTM also records the number of the channel (ch) or input (l) associated with the photon or reference event. In our work, the channel for the photon event describes the spatial signature of the detected photon, as it represents the element of the SPAD array detector which collected the photon.

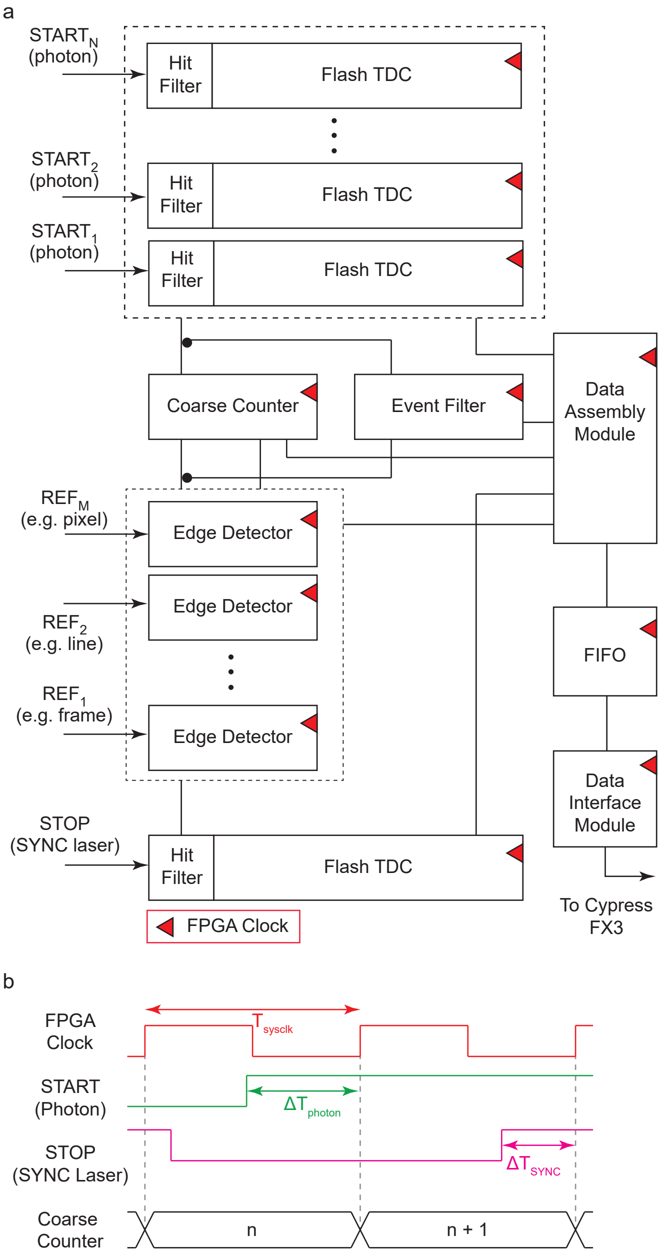

Fig. S16. Interpolating TTM-TDC FPGA architecture and general fundamental concept of the sliding scale approach. **a** Simplified architecture of the FPGA-based BrightEyes-TTM. Tapped delay line-based flash TDC modules and relative hit-filters for sampling the START_N (N = 25) signals with picosecond precision with respect to the FPGA clock (FPGA clock in red) (top portion of the figure). Free running coarse counter for implementing the sliding scale TDC technique and for sampling the REF_M (M = 3) synchronisation signals with nanosecond precision (~4.2 ns). Event-filter circuit to reduce the data throughput by transmitting information only when photons are detected (middle-top portion). Edge detector components for sampling external reference signals with nanosecond precision (i.e. pixel, line, frame clock of an imaging SP-LSM system) using a counter-based coarser TDC approach (middle-bottom portion). Single tapped delay line (hit-filter & flash TDC module) for acquiring (with picoseconds precision) the STOP signal shared by all the START_N inputs (bottom). Data assembly module for collecting a multiplicity of input digital signals and values ($\Delta T_\text{START}(ch)$ and $n_\text{photon}(ch)$, valid arrival flags for the photons, ΔT_STOP and n_SYNC and its valid digital flag for the laser sync, and the valid arrival flags for the REF_M signals) and arranging the digital data tags into a suitable form in order to be stored into a FIFO memory. FIFO memory to buffer incoming data waiting to be sent over a host-processing unit via the data interface module through the Cypress FX3 chip (right). **b** TTM interpolating architecture working principle. FPGA clock used (i) to drive all the TTM architecture components and (ii) as a fundamental reference signal for all the time measurements (top). Representation of how the TTM circuit architecture tags the START-photon (green) and STOP-sync (pink) signals with respect to the FPGA clock while computing and saving both the ΔT_START and ΔT_SYNC (i.e. integer values representing the number of tapped-delays that the signals have travelled in the delay line before the arrival of the FPGA clock rising-edge). Coarse free-running counter increasing its value at each FPGA clock rising-edge event for reconstructing, in a post-processing phase, the photon start-stop time Δt.

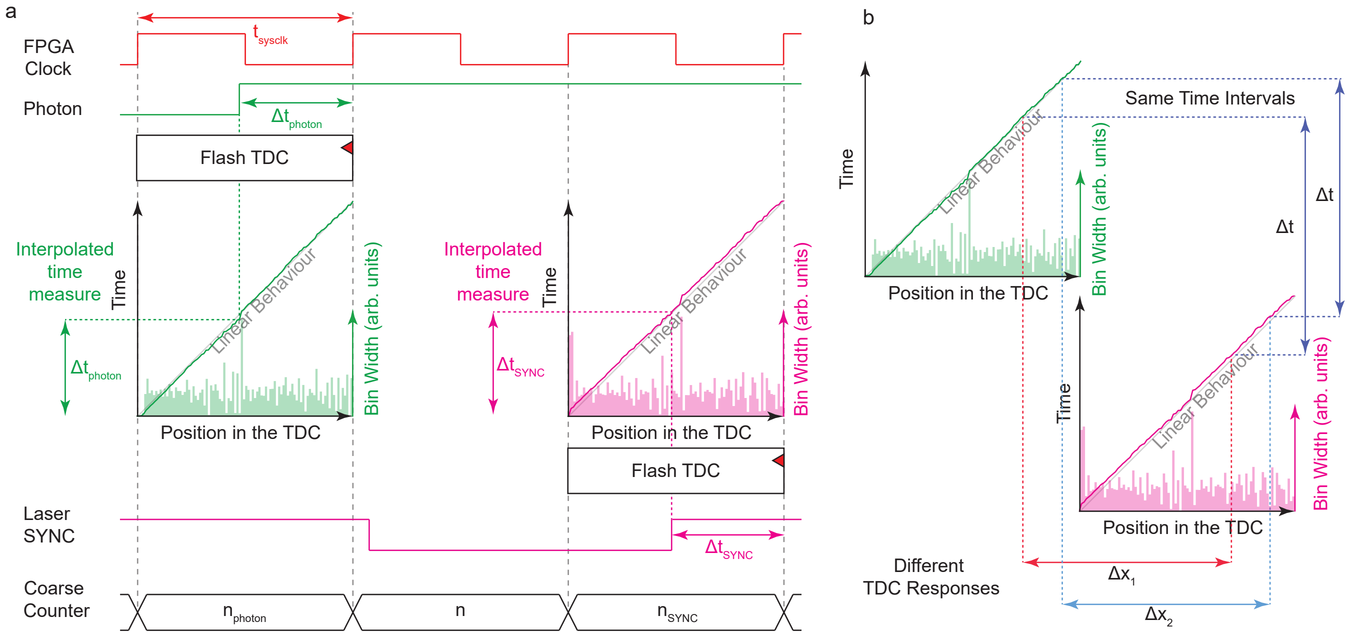

Fig. S17. Schematic of the TTM sliding scale technique working principle and mapping of $\Delta t(ch)$ start-stop time across START$_N$ and STOP$_{SYNC}$ tapped-delay-lines. **a** The START$_N$ arrival time ($\Delta t_{\text{START}}(ch)$) is computed with respect to the rising-edge of the FPGA clock (top) thanks to a dedicated flash-TDC module. Here, the time response and the tapped-delay-line bin widths are shown as a function of the bin number (left-middle). As soon as a START$_N$ is detected, also the value $n_{\text{photon}}(ch)$ of the coarse counter is registered (bottom). The STOP$_{SYNC}$ arrival time (Δt_{SYNC}) is recorded on a dedicated flash-TDC module (time response and bin-widths are show in the right-bottom portion), with respect to the FPGA clock, together with the corresponding value of the coarse counter n_{SYNC} (bottom). $\Delta t_{\text{START}}(ch)$, $n_{\text{photon}}(ch)$, Δt_{SYNC} and n_{SYNC} are used to compute $\Delta t(ch)$ (i.e. the start-stop time) according to Suppl. Eq. S1. **b** Smoothing of the flash-TDC non-linearity: because the START$_N$ and STOP$_{SYNC}$ signals are asynchronous with respect to the FPGA clock, the same start-stop time interval Δt, (i.e. the delay between a photon and a laser sync event) is measured at different positions in the flash TDC modules.

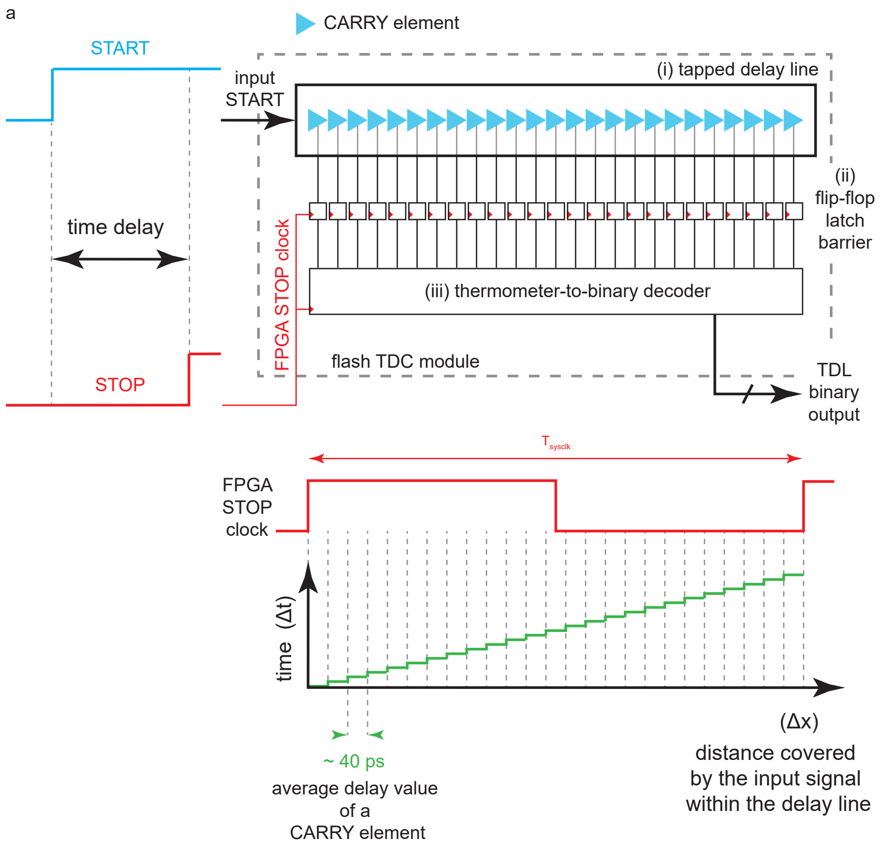

b

thermometer code	binary code
0000000	000
0000001	001
0000011	010
0000111	011
0001111	100
0011111	101
0111111	110
1111111	111

Fig. S18. Architecture of the flash time-to-digital (TDC) converter and functioning of the thermometer-to-binary encoder. **a** Schematic representation of a delay line TDC constituted by (i) a tapped-delay line composed of built-in FPGA CARRY-delay elements, (ii) a flip-flop latch barrier to sample and stabilise the tapped-delay-line readings (i.e. $\Delta T_{\text{START}}(ch)$ or ΔT_{STOP}) and (iii) a thermometer-to-binary encoder to translate the tapped-delay-line readings into a binary form (top); working principle of a TDL-TDC: the input signal propagates along the TDL until a reading event occurs. By knowing the reciprocal relationship between the CARRY delay value and the distance the input signal covered before the STOP event occurred, it is possible to convert the digital measurement of the travelled distance into a time measurement (bottom). **b** Example of thermometer-to-binary working principle: tapped-delay-line readings are converted into a binary number for a more compact and efficient data representation.

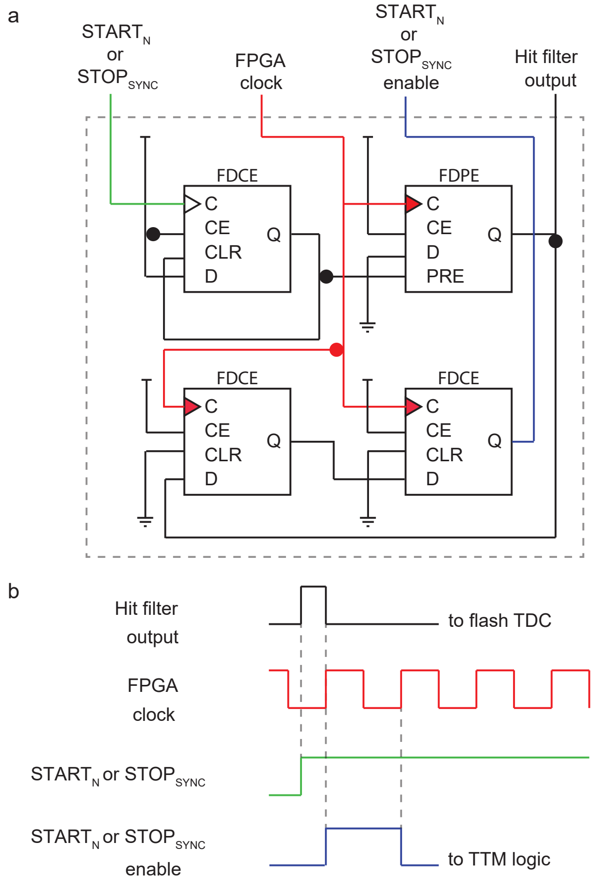

Fig. S19. Schematic circuit of the hit filter component and related input-output digital signals. **a** Hit-filter FPGA digital circuit. The digital-electronic layout of the hit-filter is made by four flip-flops. The hit-filter component is engineered to shape the incoming photons and sync signal lengths based on the FPGA clock period and, at the same time, to generate a toggle signal event (the photon or sync enable signal) for each detected $START_n$ and STOP event. The hit-filter logic is also necessary to avoid the clogging of the flash TDC module and to allow the TDC module to be ready to sample incoming signals, thus reducing the dead-time of the architecture. Thanks to the hit-filter, the architecture is independent from the signal pulse duration (hold-off). While the hit filter output signal is solely used to activate the flash TDC module, the photon enable signal is distributed to the entire TTM logic to sample and record the arrival of a photon (or sync pulse) on a specific channel. **b** Input and output signals of the hit-filter logic. Primary digital hit-filter output (top), FPGA clock (second row), rising edge of the photon or sync event (third row), photon or sync enable signal that has the duration of an FPGA clock period (bottom).

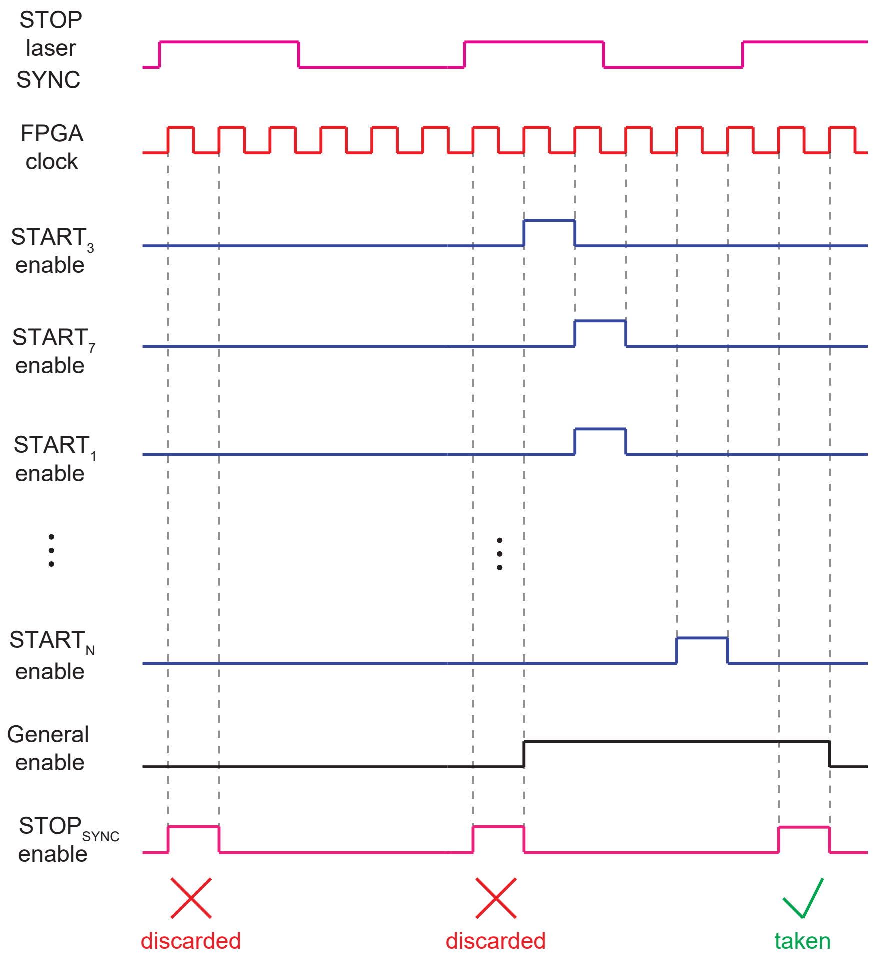

Fig. S20. Logic circuit of the event filter. All valid-enable photon signals (from *START₁ enable* to *START$_N$ enable*), produced by the hit-filter modules whenever a START-photon (or STOP$_{\text{SYNC}}$) event is detected, are used to efficiently record the multi-channel data tags ($\Delta T_{\text{START}}(ch)$ and ΔT_{STOP}). The event filter was designed to optimally handle time-tag data for high laser repetition rates (up to 80 MHz - Fig. S4). It is not efficient to sample and tag all the incoming laser pulses (laser SYNC events) and stream the related information together with the data tags ($\Delta T_{\text{START}}(ch)$, ΔT_{STOP}, $n_{\text{photon}}(ch)$, n_{SYNC}) of the photons to a processing unit. For this reason, the event filter circuit works backwards: when a photon is detected in channel i, the corresponding *START$_i$ enable* activates a general enable signal. The general enable signal remains active until the detection of a successive laser pulse sync signal (*STOP$_{SYNC}$ enable*). If the general enable signal is high at the moment of a STOP$_{\text{SYNC}}$ pulse, at least one photon has been detected and only in this case the FPGA circuit tags and registers the START-STOP times of all photons in all channels. By discarding STOP$_{\text{SYNC}}$ events that do not have a corresponding *START$_i$ enable* event, the data rate can be significantly reduced.

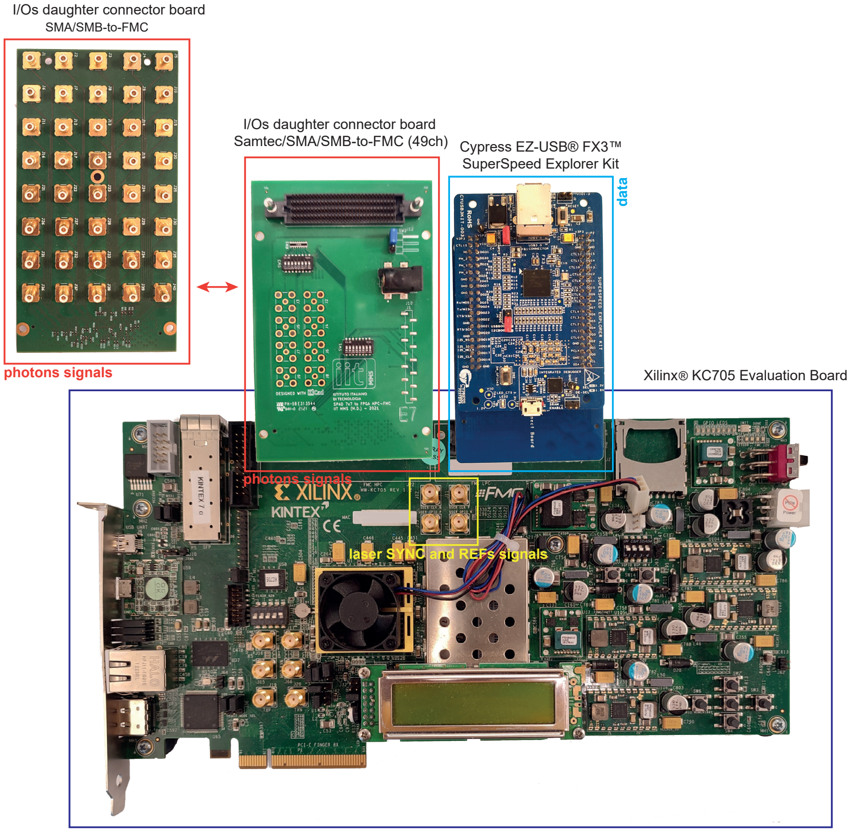

Fig. S21. BrightEyes-TTM final assembly.The Xilinx® KC705 Evaluation board, the Cypress® FX3™ SuperSpeed Explorer Kit and the daughter connector card can be easily stacked together with FMC connectors that interlock the three components. The REFs signals (e.g., pixel, line, frame clocks) and the laser SYNC signal are directly connected to the main board with dedicated SMA connectors. We developed two different daughter connector cards to match the request from the SPAD array detector. The first card developed for the 5×5 SPAD array detector prototype allows connecting the TTL photon signals using 25 SMB/SMA connectors. The second card developed for the commercial 7×7 SPAD array detector allows connecting the LVDS photon signals using a single Samtec P/N SEAF-30-05.0-L-08-2-A-LP-K-TR connector. In both cases, an extra SMA/SMB connector allows an extra input/output signal, e.g., for the duplicated signal of the SPAD central element.

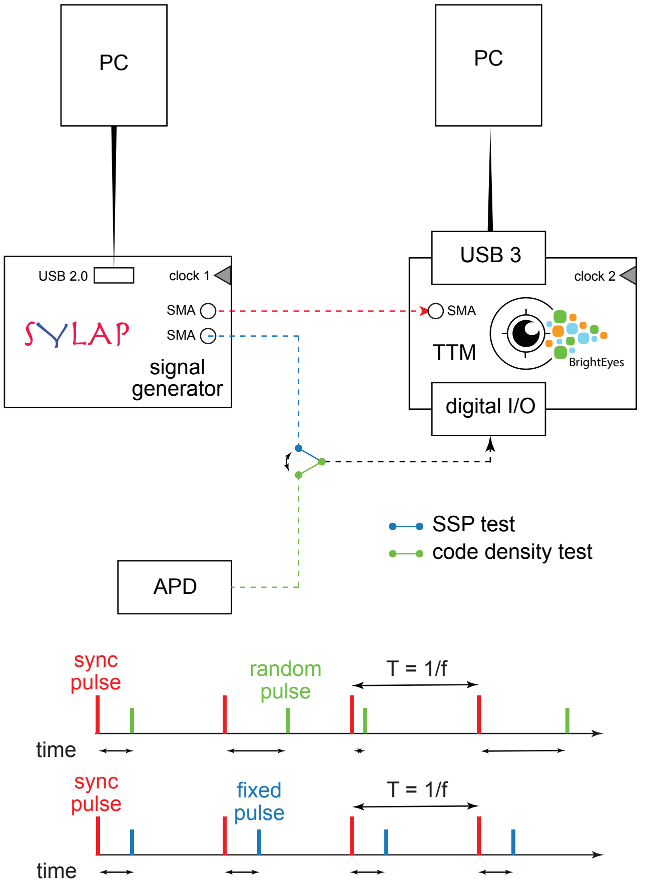

Fig. S22. Test Bench System. The setup used for the TTM tests and characterisations. The TTM STOP is connected to the clock generated by SYLAP. The TTM start input can be connected to an APD for generating asynchronized pulses (green) or can be connected to SYLAP to generate pulses with a fixed delay with respect to the clock (blue).

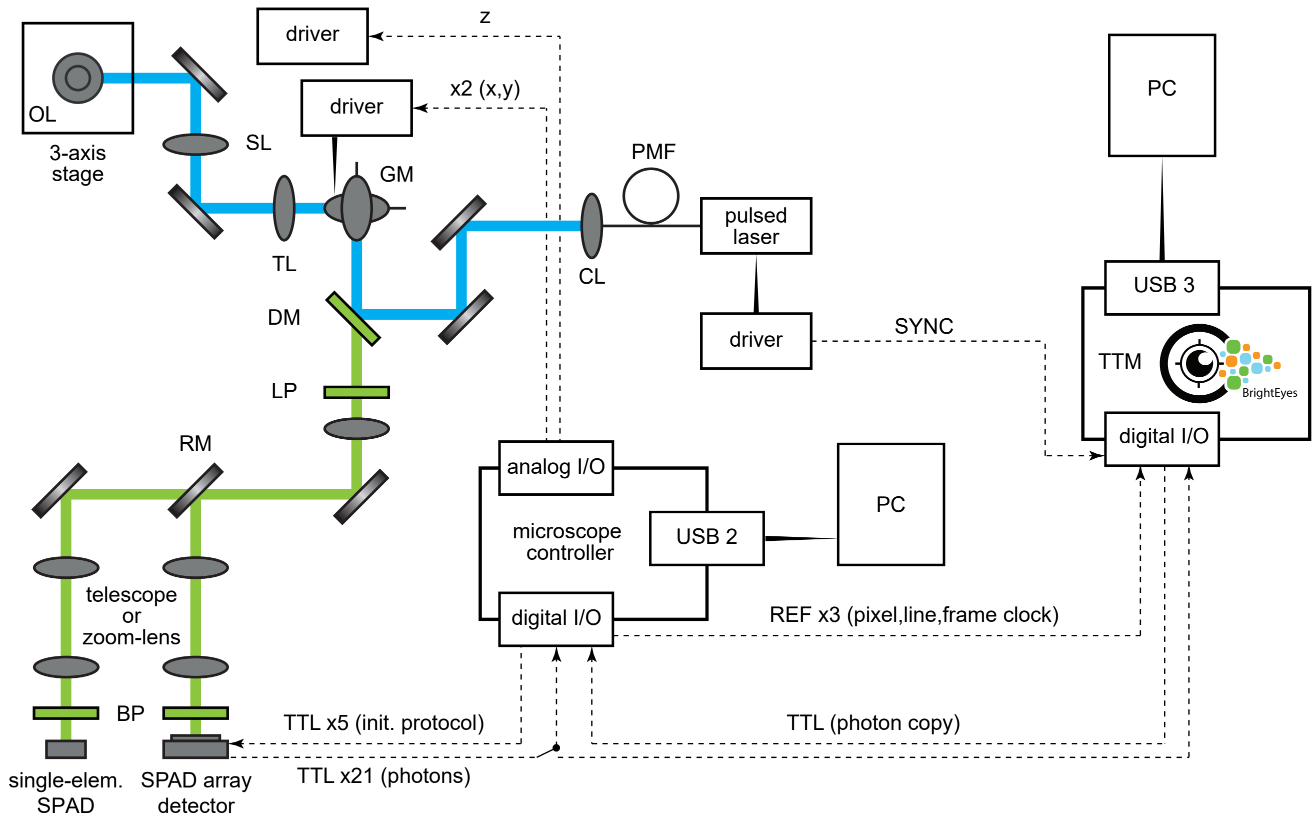

Fig. S23. Custom single-photon laser scanning microscope with a 5×5 SPAD array detector prototype. Schematic representation of the optical architecture, the data-acquisition and control system. DM: dichroic mirror, GM: galvanometric scan mirrors, RM: removable mirror, SL: scan lens, TL: tube lens, OL: objective lens, LP: long pass filter, BP: band pass filter, CL: collimating lens, PMF: polarising maintaining fiber, PC: personal computer. The microscope uses a 485 nm pulsed laser (LDH-D-C-485 PicoQuant, driven by a PicoQuant PDL 828 Sepia II Multichannel Picosecond Diode Laser driver). After passing through a 488/10 nm clean-up filter, the laser light was reflected by a dichroic beam splitter (ZT405/488/561/640 rpc, Chroma Technology Corporation) towards the galvanometric scanning mirrors. The scanning system was coupled to a 50 mm Leica scan lens and a 200 mm Leica tube lens system. All measurements with this setup were performed with a 100×/1.4 Leica objective lens. Focusing, axial scanning, and scouting of the sample region-of-interest were performed with a 3-axis stage (Nano-LP Series, Mad City Labs). The fluorescence signal was collected in de-scanned mode, passing through the dichroic beam splitter, a 488 nm long pass filter and a fluorescence emission filter (ET550/25, Chroma Technology Corporation). A 250 mm lens conjugated with the scan lens was installed to obtain a 1.4 Airy unit field of view on the 5×5 SPAD array detector, which meant that the detector also acted as a pinhole to remove the out-of-focus fluorescence background. Alternatively, the fluorescence could be detected with a commercial single-element SPAD ($PD-050-CTC-FC, Micro Photon Devices, Bolzano, Italy) after passing through a zoom-lens system consisting of a 200 mm and 50 mm lens, respectively. Digital and analogue single-cable connections are represented by dashed lines. The pixel, line, and frame reference signals and the laser sync signal are directly plugged into the FPGA-development board with the SMA user I/Os, whilst the photon signals are connected to the board through the I/Os daughter card. The board duplicates the photon signal from the central element of the SPAD array detector and sends it back to the microscope controller via a TTL digital signal.

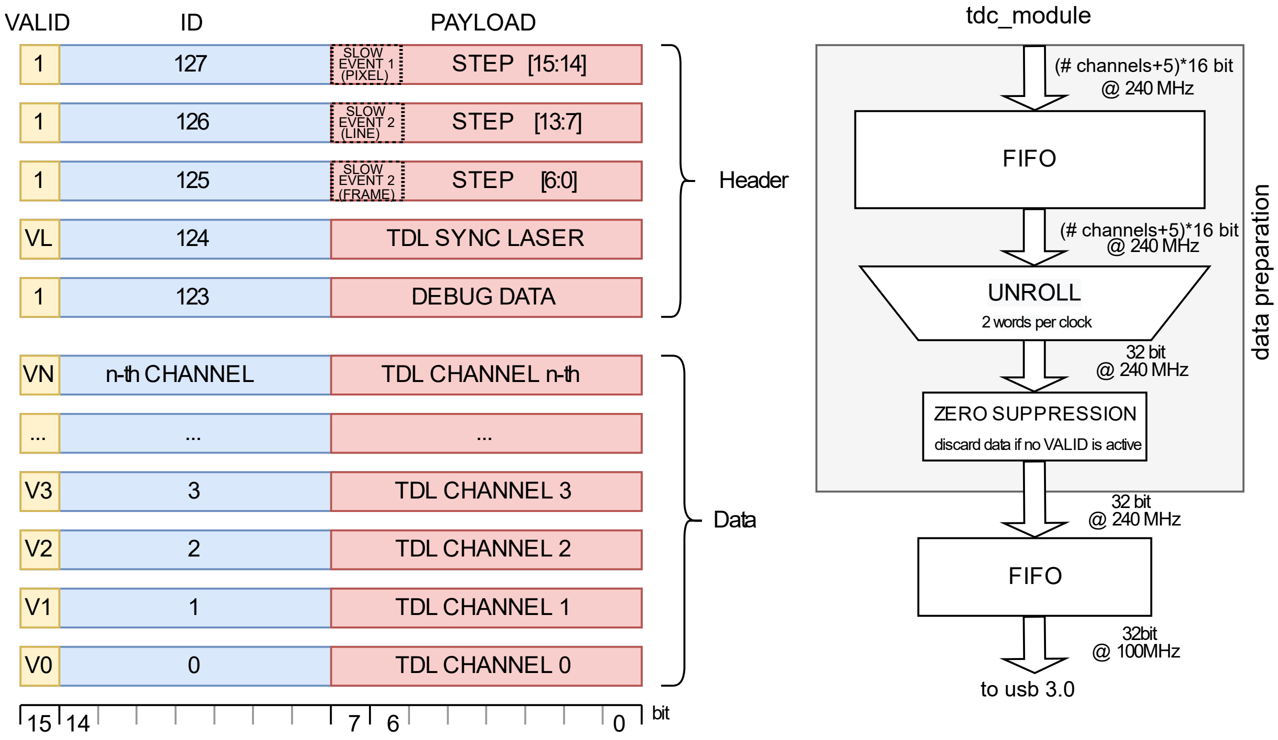

Fig. S24. Data Structure. The data structure (left) is composed of 16-bit words: 5 header words and a flexible number of words for the channels data. By design, the data structure can support up to 123 channels. The actual maximum number of channels is defined during the FPGA synthesis phase. Prior to the transmission, the data structure is processed by the data preparation module (right), which reshapes the data and joins two consecutive 16-bit words into a 32-bit word. In order to reduce the data rate, the data are only sent to the USB 3.0 module if at least one of the valid flags of the two words is active (zero-suppression).

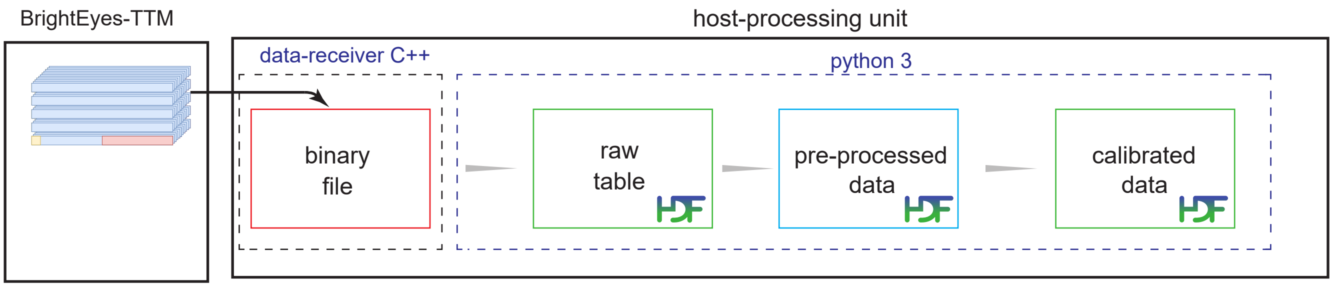

Fig. S25. Data processing pipeline. The data streamed by the BrightEyes-TTM is saved into a binary-file form using a C++ based software program. Then, the data are mapped into a raw table which gets pre-processed and refined in a python-based analysis environment for data calibration and further data reconstruction.

RAW TABLE

FREE-RUNNING COUNTER	SLOW EVENT 1 (PIXEL)	SLOW EVENT 2 (LINE)	SLOW EVENT 3 (FRAME)	SYNC (LASER) DATA VALID	TDL SYNC (LASER)	CH 1 DATA VALID	TDL CH 1	CH ... DATA VALID	TDL CH ...	CH 25 DATA VALID	TDL CH 25	
19325				1	41	1	17					1
19326										1	32	
19327						1	54					
19328								1	27			
19329				1	19							2
19330	1			1	29	1	11			1	9	3
19331			1							1	54	
19332				1	37	1	7					4
19333	1			1	13					1	8	5
…	…	…	…	…	…	…	…	…	…	…	…	

PRE-PROCESSED DATA SAVED IN SEPARATED TABLE

MAIN

idx	x	y	fr	n	ΔT_{STOP}
1	34	54	3	19325	41
2	34	54	3	19329	19
3	35	54	3	19330	29
4	0	0	4	19332	37
5	1	0	4	19333	13
	…	…	…	…	…

CH1

idx	Δn	ΔT_{START}
1	0	17
2	2	54
3	0	11
4	0	7
	…	…

CH…

idx	Δn	ΔT_{START}
2	1	27
	…	…

CH25

idx	Δn	ΔT_{START}
2	3	32
3	0	9
4	1	54
5	0	8
	…	…

CALIBRATED DATA SAVED IN SEPARATED TABLE

MAIN

idx	x	y	fr	t_{SYNC} [ps]
1	34	54	3	80520833.3
2	34	54	3	80537500.0
3	35	54	3	80541666.7
4	0	0	4	80550000.0
5	1	0	4	80554166.7
	…	…	…	…

CH1

idx	Δt [ps]
1	1152.0
2	6653.3
3	864.0
4	1440.0
	…

CH…

idx	Δt [ps]
2	3782.7
	…

CH25

idx	Δt [ps]
2	11876.0
3	960.0
4	3350.7
5	240.00
	…

Fig. S26. Data pre-processing. The table on top is the representation in table form of the binary data as received. The grey cells are the data-valid flags. Each SYNC data-valid flag (in red) is associated with a unique index (highlighted yellow number). The tables in the middle (main and channels tables) represent the data after the pre-processing. Notably, the channels tables have not all indexes, as they contain valid events only. The tables at the bottom represent the calibrated data.

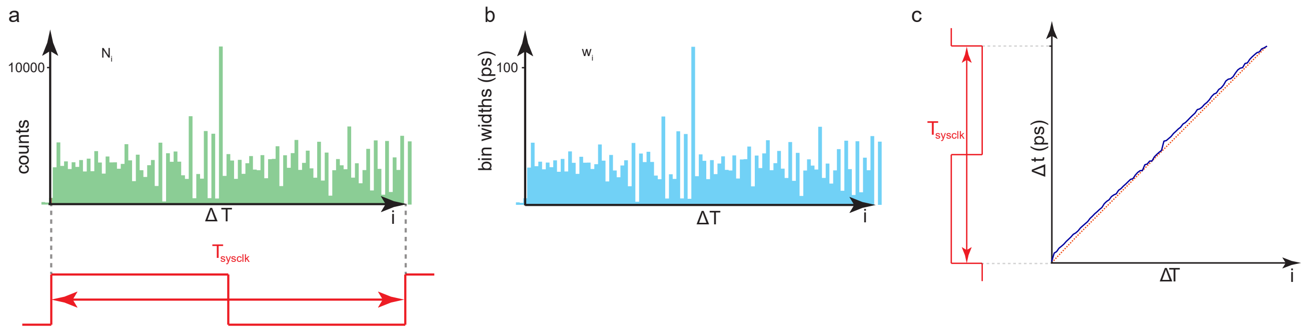

Fig. S27. Main steps of the bin-by-bin calibration procedure. **a** Tapped-delay-line histogram of the received counts as a function of the arrival tap (delay) number. The range of the maximum arrival tap number depends on the reference FPGA clock period $\mathcal{T}_{\text{sysclk}}$: the length of the tapped-delay-line is equal to the total delay value of $\mathcal{T}_{\text{sysclk}}$. **b** Estimation of the value w_i of each delay element. **c** Time as a function of ΔT: each w_i is used to compute the $\Delta t_{\text{START}}(ch)$ or Δt_{SYNC}

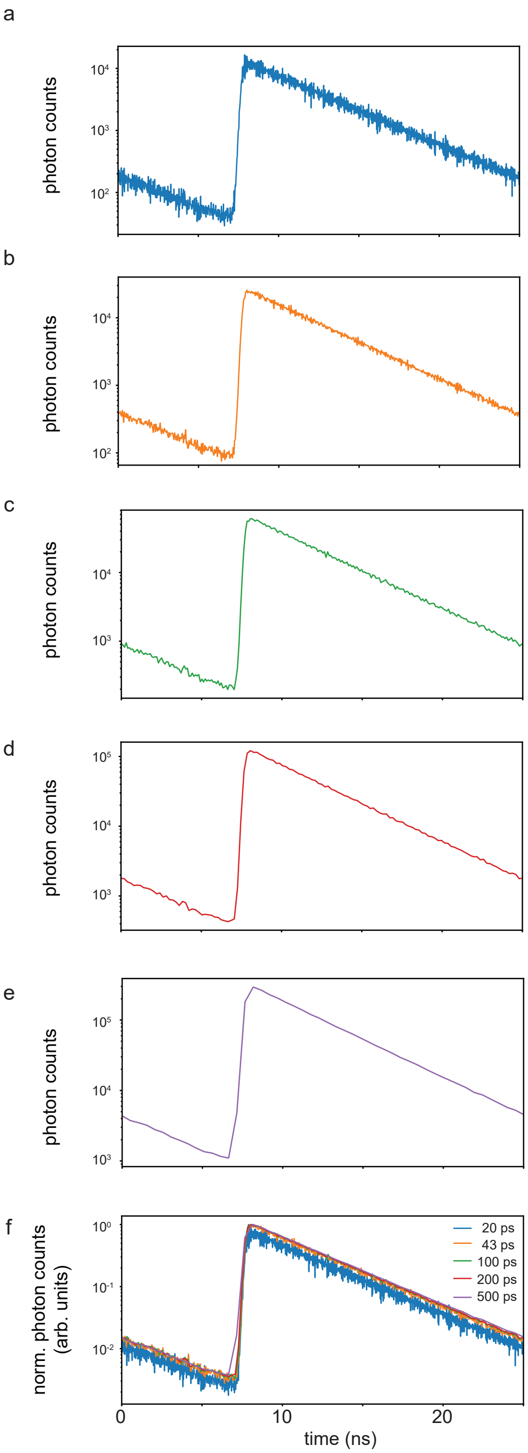

Fig. S28. TCSCP histograms of a fluorescein solution reconstructed using different time bin widths and superposition of the obtained results. Fluorescence decay histogram (photon counts as a function of time) of a fluorescein-water solution reconstructed with bin-width of **a** 20 ps, **b** 43 ps, **c** 100 ps, **d** 200 ps and **e** 500 ps. **f** cumulative view (normalised photon counts versus time) of the reconstructed fluorescein decay histograms for all the different tested time bin-widths. The 20 ps curve shows a noisier profile when compared to 43, 100, 200 and 500 ps curves, due to an undersampling of the actual bin-width for the reconstructed TCSPC histograms. Setup: custom-built single-photon laser scanning microscope equipped with a 5×5 SPAD array detector prototype. Data from central element only.

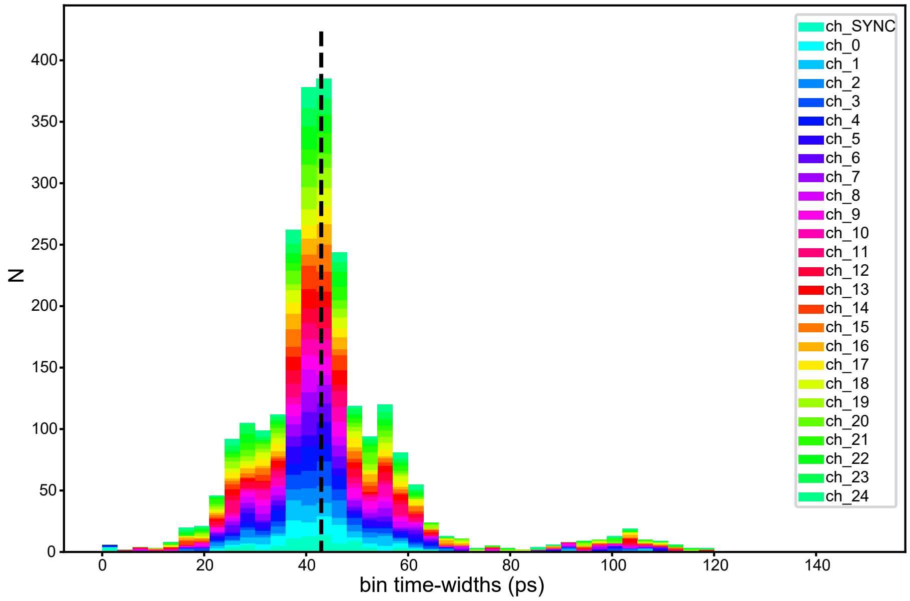

Fig. S29. Histogram of the time-bin widths (w_i) of all the deployed TDLs in the TTM architecture. Cumulative histogram, number (N) as a function of time-bin widths, of all the calculated w_i for all the TTM delay lines. The histogram mean value (red line) is w_{avg} = (43 ± 16) ps.

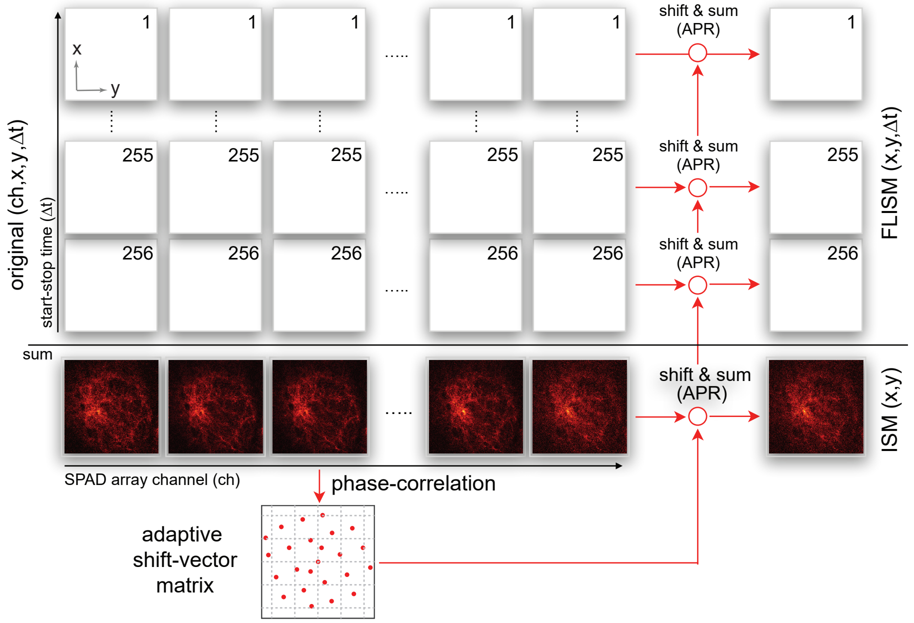

Fig. S30. Adaptive pixel-reassignment method for 4D lifetime data-set. In case of imaging, the BrightEyes-TTM provides a 4D (ch, x, y, Δt) pre-processed data-set, which can be used to reconstruct the high-resolution FLISM image. The reconstruction consists in the following steps: (i) the algorithm integrates the data-set along the start-stop Δt dimension, and generates a 3D (ch, x, y) data-set; (ii) a phase-correlation registration algorithm uses the conventional intensity data-set to calculate the shift-vector fingerprint $(s_x(ch), s_y(ch))$; (iii) for each Δt, the images (x, y) associated with each channel ch are shifted according to the shift-vector fingerprint, and summed along the ch dimension. The result is a 3D data-set (x, y, Δt) which can be used to calculate the lifetime map or the phasor-plot histogram.

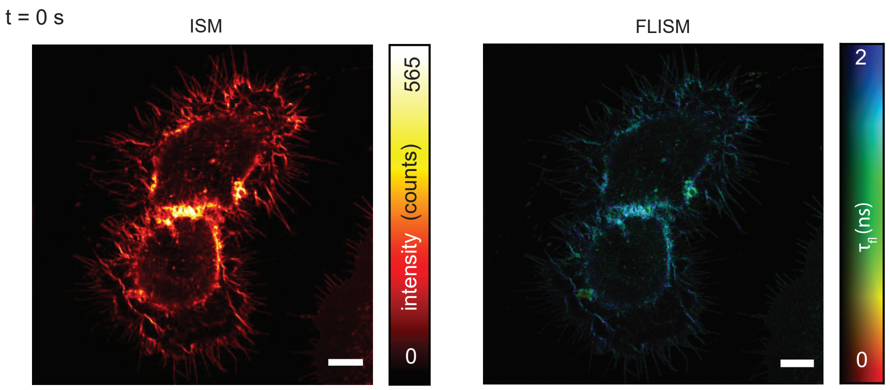

Fig. S31. Time-lapse ISM and FLISM images of a Hela cell with a membrane stain dye. Sample: Hela cell stained with the fluorescent polarity-sensitive membrane dye di-4-ANEPPDHQ. This probe allows monitoring the ordered/disordered-phase membrane domains as its fluorescence lifetime is sensitive to these changes. On the right, reconstructed ISM images and on the left super-resolved FLIM images over time. Each image is acquired every 5 minutes for over 1 hour. Scale bars 10 µm. Pixel dwell time 200 µs. The time-lapse experiment was independently performed once. Setup: custom-built single-photon laser scanning microscope equipped with a 5×5 SPAD array detector prototype and a picosecond pulsed diode laser.

Table S1. Notations

$\Delta T_{\text{START}}(ch)$	integer value representing the number of tapped-delays (in the START tapped-delay line) that the START signal, i.e., the photon signal, travels through before the arrival of the FPGA clock signal. When reported, ch denotes the photon channel.
$\Delta t_{\text{START}}(ch)$	calibrated temporal value representing the time elapsed from the START signal, i.e., the photon signal, and the next active edge of the free-running coarse counter (FPGA clock). When reported, ch denotes the photon channel.
ΔT_{STOP}	integer value representing the number of tapped-delays (in the STOP tapped-delay line) that the STOP signal, i.e., the laser SYNC signal, travels through before the arrival of the FPGA clock signal.
Δt_{STOP}	calibrated temporal value representing the time elapsed from the STOP signal, i.e., the laser SYNC signal, and the next active edge of the free-running coarse counter (FPGA clock).
$\Delta t(ch)$	photon start-stop time, i.e., the photon arrival-time in reference to the excitation event. When reported, ch denotes the photon channel.
$\hat{t}_{\text{photon}}(ch)$	photon-arrival time with respect to the beginning of the experiment measured with nanosecond precision (by using only the free-running coarse counter). When reported, ch denotes the photon channel.
$\hat{t}_{\text{SYNC}}(ch)$	SYNC-arrival time with respect to the beginning of the experiment measured with nanosecond precision (by using only the free-running coarse counter).
$\hat{t}_{\text{REF}}(l)$	REF-arrival time with respect to the beginning of the experiment measured with nanosecond precision (by using only the free-running coarse counter).
$\Delta\hat{t}^{\,\text{photon}}_{\,\text{REF}_l}(ch)$	photon-arrival time with respect to the l-th reference event measured with nanosecond precision (by using only the free-running coarse counter). When reported, ch denotes the photon channel.
$n_{\text{photon}}(ch)$	the number of FPGA clock cycles between the beginning of the experiment and the photon signal. When reported, ch denotes the photon channel.
n_{SYNC}	the number of FPGA clock cycles between the beginning of the experiment and the laser SYNC signal.
n_{REF_l}	the number of FPGA clock cycles between the beginning of the experiment and the l-th reference signal.
$\Delta n(ch)$	the number of elapsed FPGA clock cycles from the START photon event to the relative STOP laser SYNC event. When reported, ch denotes the photon channel.
$\mathcal{T}_{\text{sysclk}}$	the period of the FPGA system clock.
f_{sysclk}	frequency of the FPGA system clock.
$t_{\text{photon}}(ch)$	absolute time from the beginning of the experiment for the photon signal with, in principle, picosecond precision.
t_{SYNC}	absolute time from the beginning of the experiment for the SYNC signal with, in principle, picosecond precision.
(x, y, z)	spatial coordinate for the scanning system.
fr	frame.
τ_{fl}	fluorescence excited-state lifetime, i.e., average time that a fluorophore spends in the excited state. Assuming a single exponential decay it represents the inverse of the decay rate.
$\mathcal{T}$	the period of the pulsed excitation laser.
f	the frequency of the pulsed excitation laser.
τ_D	diffusion (or transient) time, in the context of FFS.
D	diffusion coefficient, in the context of FFS.
ω_0	detection volume lateral size, in the context of FFS .

Table S2. Comparison BrightEyes-TTM with other non-commercial Xilinx Kintex 7 FPGA-based platforms

Reference	Time bin [ps]	Single-shot precision (σ) [ps]	DNL (min:max) [LSB]	INL (min:max) [LSB]	Dead-time [ns]	Time range [ns]	Input channels
**BE-TTM**	48	30	[-0.16:0.17]	[-0.18:0.22]	$\sim$ 4.2	not limited by hardware	49
(12)	4.7	26	[-0.96:20.93]	[-15.14: 31.54]	$\sim$ 1.429	3.83E+08	1
(13)	1.3	4.6	[-0.99:4.79]	[-14.18:13.16]	8	-	2
(14)	43	34	[-0.98:0.08]	[-1:0.97]	50	-	8
(15)	11	15	[-1:1.4]	[-1.75:3.5]	-	-	32
(16)	1.87	2.79	[-0.54:+1.3]	[-2.21:3.51]	8	-	1
(17)	1.29	3.54	[-1.2:1.4]	[-3.28:3.78]	-	-	1
(18)	78.13	35	[-0.28:0.53]	[-0.56:0.38]	< 50	-	2
(19)	22.7	85.7	<3	<4	30	5.24E+03	24
(20)	8.6	20	-	-	200	1E+10	8
(5)	8.7	-	[0:4.6]	-	1.47	360	2
(21)	17.6	15	[-1:0.8]	$\pm$ 0.8	-	-	1
(22)	1.28	3.1	7	-	-	20	1
(23)	1.9	4.5	[-1:4.2]	[-12:5.8]	87.7	2.62E+14	8
(24)	23	11	[-1:2.9]	[-1:4.73]	-	8.0E+9	1
(25)	2.45	3.9	[-1:5.5]	18.8	3.6	1.183E+05	1
(26)	3.17	4.3	4.4	[-1.7:2.1]	-	50	1

Table S3. Comparison BrightEyes-TTM with other commercial FPGA-based platforms and with the Becker&Hickl DPC230

Parameter	BrightEyes-TTM	MultiHarp 160[1]	Time Tagger Ultra[2]	Time Tagger X[2]	Becker&Hickl DPC230[3]
**Single-shot precision (σ)**	30 ps	< 45 ps	42 ps	2 ps	-
**Time range**	Not limited by hardware	328 ns to 2.74 s	-	-	-
**Maximum laser sync rate**	80 MHz	1.2 GHz	475 MHz	700 MHz	150 MHz
**Dead time**	About 4.2 ns	< 650 ps	2.1 ns	1.5 ns	< 10 ns
**Differential non-linearity**	6% RMS	< 10% peak, < 1% RMS (over the full measurement range)	-	-	-
**Input channels (per standalone module)**	25 inputs (upgradable to 49 inputs) with 30 ps precision for sampling photons, 1 laser sync input, 3 synchronization inputs for low res sampling	16 inputs with < 45 ps precision for sampling photons, 1 laser sync input	18 inputs with 42 ps precision for sampling photons, 1 laser sync input	18 inputs with 2 ps precision for sampling photons 1 laser sync input	16 inputs, 1 laser sync input, 3 synchronization inputs for low resolution sampling

1 https://www.picoquant.com/products/category/tcspc-and-time-tagging-modules/multiharp-160#specification
2 https://www.swabianinstruments.com/time-tagger/
3 https://www.becker-hickl.com/products/dpc-230/

Supplementary References

1. Bayer, E. & Traxler, M. A high-resolution (< 10 ps RMS) 48-channel time-to-digital converter (TDC) implemented in a field programmable gate array (FPGA). *IEEE Trans. Nucl. Sci.* **58**, 1547–1552 (2011).
2. Tontini, A., Gasparini, L., Pancheri, L. & Passerone, R. Design and characterization of a low-cost FPGA-based TDC. *IEEE Trans. Nucl. Sci.* **65**, 680–690 (2018).
3. Tancock, S., Arabul, E. & Dahnoun, N. A review of new time-to-digital conversion techniques. *IEEE Trans. Instrum. Meas.* **68**, 3406–3417 (2019).
4. Wu, J. & Shi, Z. The 10-ps wave union TDC: Improving FPGA TDC resolution beyond its cell delay. In *2008 IEEE Nuclear Science Symposium Conference Record* (IEEE, 2008).
5. Liu, C. & Wang, Y. A 128-channel, 710 m samples/second, and less than 10 ps RMS resolution time-to-digital converter implemented in a Kintex-7 FPGA. *IEEE Trans. Nucl. Sci.* **62**, 773–783 (2015).
6. Carra, P. *et al.* Auto-calibrating TDC for an SoC-FPGA data acquisition system. *IEEE Trans. Radiat. Plasma Med. Sci.* **3**, 549–556 (2019).
7. Villa, F. *et al.* CMOS SPADs with up to 500 μm diameter and 55% detection efficiency at 420 nm. *J. Mod. Opt.* **61**, 102–115 (2014).
8. Klär, H., Schulz, M., Steffen, P. & Düllmann, D. The flash-TDC. *Nucl. Instrum. Methods Phys. Res. A* **275**, 197–198 (1989).
9. Parsakordasiabi, M., Vornicu, I., Rodríguez-Vázquez, Á. & Carmona-Galán, R. A low-resources TDC for multi-channel direct ToF readout based on a 28-nm FPGA. *Sensors* **21**, 308 (2021).
10. Favi, C. & Charbon, E. A 17ps time-to-digital converter implemented in 65nm FPGA technology. In *Proceeding of the ACM/SIGDA international symposium on Field programmable gate arrays - FPGA '09* (ACM Press, 2009).
11. Homulle, H., Regazzoni, F. & Charbon, E. 200 MS/s ADC implemented in a FPGA employing TDCs. In *Proceedings of the 2015 ACM/SIGDA International Symposium on Field-Programmable Gate Arrays* (ACM, 2015).
12. Kwiatkowski, P. Employing FPGA DSP blocks for time-to-digital conversion (2019).
13. Mao, X. *et al.* A low temperature coefficient time-to-digital converter with 1.3 ps resolution implemented in a 28 nm FPGA. *Sensors* **22**, 2306 (2022).
14. Zhang, J., Wang, Y. & Song, Z. A ring-oscillator based multi-mode time-to-digital converter on Xilinx Kintex-7 FPGA. *Nucl. Instrum. Methods Phys. Res. A* **1011**, 165578 (2021).
15. Tang, Y. *et al.* A highly linear FPGA-based TDC and a low-power multichannel readout ASIC with a shared SAR ADC for SiPM detectors. *IEEE Trans. Nucl. Sci.* **68**, 2286–2293 (2021).
16. Deng, J. *et al.* A tunable parameter, high linearity time-to-digital converter implemented in 28-nm FPGA. *IEEE Trans. Instrum. Meas.* **70**, 1–12 (2021).
17. Zhang, M. *et al.* High-resolution time-to-digital converters implemented on 40-, 28-, and 20-nm FPGAs. *IEEE Trans. Instrum. Meas.* **70**, 1–10 (2021).
18. Dong, X., Ma, C., Zhao, X., Li, X. & Huang, Z. A high resolution multi-phase clock time-digital convertor implemented on Kintex-7 FPGA. *J. Instrum.* **15**, T11005–T11005 (2020).
19. Torres, J. *et al.* Time-to-digital converter based on FPGA with multiple channel capability. *IEEE Trans. Nucl. Sci.* **61**, 107–114 (2014).
20. Lusardi, N. & Geraci, A. 8-channels high-resolution TDC in FPGA. In *2015 IEEE Nuclear Science Symposium and Medical Imaging Conference (NSS/MIC)* (IEEE, 2015).
21. Wang, Y. & Liu, C. A nonlinearity minimization-oriented resource-saving time-to-digital converter implemented in a 28 nm Xilinx FPGA. *IEEE Trans. Nucl. Sci.* **62**, 2003–2009 (2015).
22. Cao, Q., Wang, Y. & Liu, C. A combination of multiple channels of FPGA based time-to-digital converter for high time precision. In *2016 IEEE Nuclear Science Symposium, Medical Imaging Conference and Room-Temperature Semiconductor Detector Workshop (NSS/MIC/RTSD)* (IEEE, 2016).
23. Szplet, R., Kwiatkowski, P., Jachna, Z. & Rozyc, K. An eight-channel 4.5-ps precision timestamps-based time interval counter in FPGA chip. *IEEE Trans. Instrum. Meas.* **65**, 2088–2100 (2016).
24. Yao, Y., Wang, Z., Lu, H., Chen, L. & Jin, G. Design of time interval generator based on hybrid counting method. *Nucl. Instrum. Methods Phys. Res. A* **832**, 103–107 (2016).
25. Wang, Y., Kuang, J., Liu, C. & Cao, Q. A 3.9-ps RMS precision time-to-digital converter using ones-counter encoding scheme in a Kintex-7 FPGA. *IEEE Trans. Nucl. Sci.* **64**, 2713–2718 (2017).
26. Wang, Y., Cao, Q. & Liu, C. A multi-chain merged tapped delay line for high precision time-to-digital converters in FPGAs. *IEEE Trans. Circuits Syst. II Express Briefs* **65**, 96–100 (2018).
