# Supplementary material for: The BrightEyes-TTM as an open-source time-tagging module for democratising single-photon microscopy
Source: Nat Commun. 2022 Dec 1;13:7406. doi: 10.1038/s41467-022-35064-0 (PMC9715684; doi:10.1038/s41467-022-35064-0)
Supplement: Supplementary file 3 — Description of Additional Supplementary Files [file 41467_2022_35064_MOESM3_ESM.pdf]

Description of Additional Supplementary Files

Supplementary Movie 1: Time-lapse ISM and FLISM images of a Hela cell with a membrane stain dye. Sample: Hela cell stained with the fluorescent polarity-sensitive membrane dye di4-ANEPPDHQ. This probe allows monitoring the ordered/disordered-phase membrane domains as its fluorescence lifetime is sensitive to these changes. On the right, reconstructed ISM images and on the left super-resolved FLIM images over time. Each image is acquired every 5 minutes for over 1 hour. Scale bars 10 μm. Pixel dwell time 200 μs. Setup: custom-built single-photon laser scanning microscope equipped with a 5×5 SPAD array detector prototype and a picosecond pulsed diode laser
