# Supplementary material for: The BrightEyes-TTM as an open-source time-tagging module for democratising single-photon microscopy
Source: Nat Commun. 2022 Dec 1;13:7406. doi: 10.1038/s41467-022-35064-0 (PMC9715684; doi:10.1038/s41467-022-35064-0)
Supplement: Supplementary file 5 — Reporting Summary [file 41467_2022_35064_MOESM5_ESM.pdf]

nature portfolio

Corresponding author(s): Giuseppe Vicidomini

Last updated by author(s): 2022/08/31

Reporting Summary

Nature Portfolio wishes to improve the reproducibility of the work that we publish. This form provides structure for consistency and transparency in reporting. For further information on Nature Portfolio policies, see our Editorial Policies and the Editorial Policy Checklist.

Statistics

For all statistical analyses, confirm that the following items are present in the figure legend, table legend, main text, or Methods section.

n/a	Confirmed	
[ ]	[x]	The exact sample size (*n*) for each experimental group/condition, given as a discrete number and unit of measurement
[ ]	[x]	A statement on whether measurements were taken from distinct samples or whether the same sample was measured repeatedly
[x]	[ ]	The statistical test(s) used AND whether they are one- or two-sided<br>*Only common tests should be described solely by name; describe more complex techniques in the Methods section.*
[x]	[ ]	A description of all covariates tested
[x]	[ ]	A description of any assumptions or corrections, such as tests of normality and adjustment for multiple comparisons
[ ]	[x]	A full description of the statistical parameters including central tendency (e.g. means) or other basic estimates (e.g. regression coefficient) AND variation (e.g. standard deviation) or associated estimates of uncertainty (e.g. confidence intervals)
[x]	[ ]	For null hypothesis testing, the test statistic (e.g. *F*, *t*, *r*) with confidence intervals, effect sizes, degrees of freedom and *P* value noted<br>*Give P values as exact values whenever suitable.*
[x]	[ ]	For Bayesian analysis, information on the choice of priors and Markov chain Monte Carlo settings
[x]	[ ]	For hierarchical and complex designs, identification of the appropriate level for tests and full reporting of outcomes
[x]	[ ]	Estimates of effect sizes (e.g. Cohen's *d*, Pearson's *r*), indicating how they were calculated

Our web collection on statistics for biologists contains articles on many of the points above.

Software and code

Policy information about availability of computer code

Data collection	BrightEyes-TTM available through our GitHub repository https://github.com/VicidominiLab/BrightEyes-TTM<br>Python 3.0<br>Labview 2019
Data analysis	Python 3.0<br>ImageJ 1.53<br>FLIMJ plugin (ImageJ) 1.1.0<br>BrightEyes-TTM available through our GitHub repository https://github.com/VicidominiLab/BrightEyes-TTM

For manuscripts utilizing custom algorithms or software that are central to the research but not yet described in published literature, software must be made available to editors and reviewers. We strongly encourage code deposition in a community repository (e.g. GitHub). See the Nature Portfolio guidelines for submitting code & software for further information.

Data

Policy information about availability of data

All manuscripts must include a data availability statement. This statement should provide the following information, where applicable:

- Accession codes, unique identifiers, or web links for publicly available datasets
- A description of any restrictions on data availability
- For clinical datasets or third party data, please ensure that the statement adheres to our policy

As keen proponents of open science and open data, we have made the raw time-tagged data, which supports the findings of this study, publicly available at Zenodo, https://doi.org/10.5281/zenodo.4912656. Full build instructions for the BrightEyes-TTM are available through our GitHub repository https://github.com/VicidominiLab/BrightEyes-TTM

Human research participants

Policy information about studies involving human research participants and Sex and Gender in Research.

Reporting on sex and gender	N/A
Population characteristics	N/A
Recruitment	N/A
Ethics oversight	N/A

Note that full information on the approval of the study protocol must also be provided in the manuscript.

Field-specific reporting

Please select the one below that is the best fit for your research. If you are not sure, read the appropriate sections before making your selection.

☒ Life sciences ☐ Behavioural & social sciences ☐ Ecological, evolutionary & environmental sciences

For a reference copy of the document with all sections, see nature.com/documents/nr-reporting-summary-flat.pdf

Life sciences study design

All studies must disclose on these points even when the disclosure is negative.

Sample size	Regarding the imaging experiments, in particular the FLISM measurements, we consider as sample size, the number of pixels for each field-of-view acquired were calculated based on the Nyquist sampling condition while the exposure times were decided based on the intensity flux in order to minimize photo-damage and avoid saturation. Regarding the FLFS experiments, data were acquired long enough to assure enough intensity signal in order to have clear autocorrelation functions.
Data exclusions	For the FFS experiments, the individual correlation curves were visually inspected and all curves without artifacts were averaged. The correlation curves with clear artifacts were excluded.
Replication	We tested the BrightEyes-TTM on three different optical setups, two custom-built laser-scanning microscopes (one with a commercially available SPAD array detector and one equipped with a SPAD array detector prototype) and one commercial microscope (Nikon AXR confocal system). On the different setups, both imaging and spectroscopy experiments were performed. Regarding the FFS measurement of fluorescent beads, data were acquired for 220 s, divided in chunks of 10 s each and averaged before the analysis. Regarding the FLFS measurements in living cells, the data were acquired from 5 different cells, in each cell multiple positions (from 3 to 5) were sampled. The cell experiments were independently conducted twice.
Randomization	This is not relevant for all the method in the paper because they do not depend on the statistical variation of the sample. Regarding the FLFS measurements in living cells, in this biological context, no differences in the fluorescence lifetime and in the protein diffusion are expected over time, as eGFP is not specifically tagged to any protein.
Blinding	Blinding is not relevant to this study because we did not have experiments where we compared different conditions.

Reporting for specific materials, systems and methods

We require information from authors about some types of materials, experimental systems and methods used in many studies. Here, indicate whether each material, system or method listed is relevant to your study. If you are not sure if a list item applies to your research, read the appropriate section before selecting a response.

Materials & experimental systems

n/a	Involved in the study	
☐	☒	Antibodies
☐	☒	Eukaryotic cell lines
☒	☐	Palaeontology and archaeology
☐	☒	Animals and other organisms
☒	☐	Clinical data
☒	☐	Dual use research of concern

Methods

n/a	Involved in the study	
☒	☐	ChIP-seq
☒	☐	Flow cytometry
☒	☐	MRI-based neuroimaging

Antibodies

Antibodies used	Monoclonal anti-alpha-tubulin antibody (mouse IgG1 isotype), Sigma-Aldrich, T5168, clone B-5-1-2<br>Goat anti-mouse IgG highly cross-adsorbed secondary antibody Alexa Fluor 488, Sigma-Aldrich, A-11029
Validation	Any information about the validation of the primary anti-alpha-tubulin antibody is provided by the supplier (https://www.sigmaaldrich.com/IT/it/product/sigma/t5168).

Eukaryotic cell lines

Policy information about cell lines and Sex and Gender in Research

Cell line source(s)	HeLa (human adenocarcinoma epithelial cells, cervix) and HEK-293T (human embryonic epithelial cells, kidney) cell lines have been purchased by Sigma-Aldrich. Primary hippocampal mice neurons has been obtained by the dissection of postnatal mouse hippocampus.
Authentication	HeLa and HEK293T were not authenticated.
Mycoplasma contamination	HeLa and HEK-293T cell lines and primary cells were not tested for mycoplasma contamination.
Commonly misidentified lines<br>(See ICLAC register)	HEK293T cells were used but non-authenticated.

Animals and other research organisms

Policy information about studies involving animals; ARRIVE guidelines recommended for reporting animal research, and Sex and Gender in Research

Laboratory animals	In this study wild type mice B6;129-Nlgn3wt/J (B6129SF1/J) of either sex were used at postnatal day 1 to obtain primary neuronal cultures.
Wild animals	the study did not involve wild animals
Reporting on sex	Sex-based analysis were not considered, as only primary neurons were involved in the study. Moreover, in the manuscript primary cell lines were only used as proof-of-principle of our time tagging platform.
Field-collected samples	The study did not involved samples collected from the field.
Ethics oversight	The experiments involving primary neuronal cultures from mice were prepared in accordance with the guidelines established by the European Communities Council (Directive 2010/63/EU of 22 September 2010) and following the Italian law D.Lgs.26/2014. All the animal procedures have been approved by the Italian Ministry of Health Regulation (Authorization 800/2021-PR) and by the Italian Institute of Technology welfare body.

Note that full information on the approval of the study protocol must also be provided in the manuscript.
